# Genome-Wide Analysis of the Fatty Acid Desaturase Gene Family Reveals the Key Role of *PfFAD3* in α-Linolenic Acid Biosynthesis in Perilla Seeds

**DOI:** 10.3389/fgene.2021.735862

**Published:** 2021-11-24

**Authors:** Wu Duan, Yang Shi-Mei, Shang Zhi-Wei, Xu Jing, Zhao De-Gang, Wang Hong-Bin, Shen Qi

**Affiliations:** ^1^ Institute of Medical Plant Physiology and Ecology, School of Pharmaceutical Sciences, Guangzhou University of Chinese Medicine, Guangzhou, China; ^2^ Key Laboratory of Plant Resource Conservation and Germplasm Innovation in Mountainous Region, College of Life Sciences, Guizhou University, Guiyang, China; ^3^ Guizhou Rapeseed Institute, Guizhou Academy of Agricultural Sciences, Guiyang, China

**Keywords:** perilla, α-linolenic acid (C18:3), fatty acid desaturases, genome-wide analysis, plant transformation

## Abstract

Perilla (*Perilla frutescens*), a traditional medicinal and oilseed crop in Asia, contains extremely high levels of polyunsaturated α-linolenic acid (ALA) (up to 60.9%) in its seeds. ALA biosynthesis is a multistep process catalyzed by fatty acid desaturases (FADs), but the *FAD* gene family in perilla has not been systematically characterized. Here, we identified 42 *PfFADs* in the perilla genome and classified them into five subfamilies. Subfamily members of *PfFADs* had similar exon/intron structures, conserved domain sequences, subcellular localizations, and cis-regulatory elements in their promoter regions. *PfFAD*s also possessed various expression patterns. *PfFAD3.1* was highly expressed in the middle stage of seed development, whereas *PfFAD7/8.3* and *PfFAD7/8.5* were highly expressed in leaf and later stages of seed development, respectively. Phylogenetic analysis revealed that the evolutionary features coincided with the functionalization of different subfamilies of PUFA desaturase. Heterologous overexpression of *PfFAD3.1* in *Arabidopsis thaliana* seeds increased ALA content by 17.68%–37.03%. These findings provided insights into the characteristics and functions of *PfFAD* genes in perilla.

## Introduction

Fatty acids (FAs), as a major energy source of plants and animals, are the main components of membrane lipids (e.g., phospholipids) and seed storage lipids (e.g., triacylglycerols) in plants (Manan et al., 2017). α-Linolenic acid (ALA, C18:3 Δ^9,12,15^) is primarily synthesized in oil-producing algae and terrestrial plants (Ángela et al., 2015). As one of ω-3 polyunsaturated fatty acids, ALA is crucial for maintaining the fluidity of membrane lipids in plants and a precursor of FA-derived signaling molecules such as jasmonic acid, which play important roles in plant development and stress responses (Weber, 2002). ALA is also the precursor for the biosynthesis of eicosapentaenoic acid (C20:5 ^Δ5,8,11,14,17^) and docosahexaenoic acid (C22:6 Δ^4,7,10,13,16,19^), which have important health benefits for humans, such as regulating body development, promoting brain development, reducing blood pressure, and inhibiting senescence, with therapeutical effects on neurological, cardiovascular, and cerebrovascular diseases (Baker et al., 2016). Since most animals do not produce ALA, they must obtain ALA from plants in their diet. Hence, oilseed crops rich in ALA have become an important research focus in recent years (Baker et al., 2016).

Perilla (*Perilla frutescens* var. *frutescens*, 2n = 40) is an annual herbaceous plant of the Lamiaceae family (Nitta et al., 2005). Perilla originated in China and is widely cultivated in Asian countries including Japan and Korea (Park et al., 2008). As a traditional medicinal and edible plant, perilla is widely used in the pharmaceutical, healthcare, functional food, and cosmetics industries (Igarash and Miyazaki, 2013; Mungmai et al., 2020). Perilla is also an important oilseed crop with high seed oil content (40%), of which ALA accounts for 60.9% (Ciftci et al., 2012).

Polyunsaturated fatty acid (PUFA) biosynthesis is catalyzed by a series of enzymes called fatty acid desaturases (FADs) (Alonso et al., 2003). In higher plants, the first-step desaturases are catalyzed primarily by Δ7/Δ9 desaturases (Smith et al., 2013). Δ9 desaturases, the only desaturases present in all organisms are soluble acyl-acyl carrier protein desaturases (Kachroo et al., 2007). Δ12 and ω-3 desaturases are widely reported in plants. They are used as secondary and tertiary desaturases catalyzing the conversion of oleic acid (C18:1) to linoleic acid (C18:2) and further produce ALA, respectively (Bhunia et al., 2016). Front-end FADs are functionally heterologous enzymes that produce long-chain PUFAs (Meesapyodsuk and Xiao, 2012). FAD4s, a novel class of FADs, produce Δ3-desaturated FAs in plants (Gao et al., 2009; Horn et al., 2020). Three highly conserved histidine motifs are present in most FAD proteins, which are thought to be pivotal for maintaining their catalytic activities (Sperling et al., 2003; Berestovoy et al., 2020).

ω-3 Desaturases (ω-3 FADs) are catalytic enzymes for ALA biosynthesis. Three genes encoding ω-3 FADs (*FAD3*, *FAD7*, and *FAD8*) in *Arabidopsis thaliana* share over 65% sequence identity (Iba et al., 1993; Watahiki and Yamamoto, 1994). *FAD3* encodes an endoplasmic reticulum membrane-bound desaturase that mainly functions in ALA biosynthesis in seeds (Zhou et al., 2010). *FAD7* and *FAD8* encode plastid membrane-bound desaturases with non-redundant roles in plant responses to low temperature and other environment stresses (Matsuda et al., 2005; Ángela et al., 2015). ω-3 FAD orthologous genes have been cloned and characterized in diverse plant species, such as flax (*Linum usitatissimum*) (Vrinten et al., 2005), soybean (*Glycine max*) (Anai et al., 2005), and rapeseed (*Brassica napus*) (Yang et al., 2012). These enzymes play important roles in ALA accumulation in plants.

*PfFAD3* cDNA was isolated from developing perilla seeds and determined to be specifically expressed in seeds (Abdelreheem and Hildebrand, 2013). Transcriptome analysis suggested that *PfFAD3* encodes one major desaturase for seed ALA content in perilla (Zhang et al., 2017). However, the heterologous expression of *PfFAD3* in yeast resulted in limited production of ALA (1.3%) (Lee et al., 2016). Therefore, the specific function of *PfFAD3* remains unclear.

In this study, we performed genome-wide analysis and identified 42 *PfFAD* genes in the perilla genome. We also performed phylogenetic analysis of PUFA desaturase (FADs related to polyunsaturated FA synthesis) in plants*.* Transcriptomic analysis and quantitative reverse-transcription PCR (qRT-PCR) revealed the tissue-specific expression patterns of *PfFAD* genes. We cloned *PfFAD3.1*, which is highly expressed in perilla seeds, and heterologously overexpressed this gene in *A. thaliana*. The systematic identification and functional feature laid the foundation for functional research on other *PfFADs*.

## Materials and Methods

### Plant Materials

A perilla variety (*Perilla frutescens* var. *frutescens*) with high seed oil content was selected for this study. The plants were grown in a greenhouse at the Institute of Medicinal Plant Physiology and Ecology, Guangzhou University of Chinese Medicine (23°06ʹN, 113°40ʹE)*.* The hypocotyls and cotyledons of perilla were collected 10 days after seed germination; the roots, stems, and leaves of perilla were collected at the three-leaf period of seedling growth, and flowers were collected at the flowering period. Seeds of perilla were collected 5 days after flowering (DAF), 10DAF, 15DAF, and 20DAF. All tissues were immediately frozen in liquid nitrogen and stored at −80°C until use for RNA extraction and qRT-PCR analysis.

Columbia wild-type (WT) *Arabidopsis thaliana* was used for gene transformation. WT and transgenic *A. thaliana* were kept in the dark at 4°C for 3 days to break dormancy (stratification) and then moved into a climate chamber with a photoperiod of 16 h light/8 h dark at 22°C.

### Identification of *PfFAD* Gene Family Members

Two methods were used to identify *PfFAD* gene family members. For hidden Markov method (HMM) searches, an HMM file (FA_desaturase domain: PF00487; FA_desaturase_2 domain: PF03405) was downloaded from the Pfam database (http://Pfam.xfam.org/search#tabview=tab3) (Finn et al., 2016), and HMMER 3.0 was used to retrieve the FAD sequences from the genome database (Potter et al., 2018). For BLAST alignment, the *A. thaliana* FAD protein sequences (Supplementary Table S1) were download from The Arabidopsis Information Resource (version 9.0: https://www.arabidopsis.org/) and used as queries to search for perilla FADs using the BLATSP programs with an E value of 1e–10. The sequences identified using the two methods were merged, and genes lacking the intact FAD domain were removed.

The whole-genome shogun contigs (WGS) of perilla (taxid:48,385) was used to analyze the position of *PfFAD* genes (Zhang et al., 2021). Complete domain verification of the acquired perilla FADs was performed in Pfam (http://pfam.xfam.org/search#tabview=tab1). The structures of the perilla *FAD* genes were identified with the GSDS tool (http://gsds.cbi.pku.edu.cn/) based on genomic DNA sequences and coding sequences (CDSs) (Hu et al., 2014). The conserved domains and motifs of the perilla FADs were analyzed using the online software Pfam and MEME (http://meme-suite.org/) (Bailey et al., 2009). The chemical properties and subcellular localizations of the proteins were examined using ExPASy (http://web.expasy.org/protparam/) and Cell-PLoc (http://www.csbio.sjtu.edu.cn/bioinf/plant/) (Artimo et al., 2012; Chou and Shen, 2008). To analyze the promoters of the *FAD* genes, the upstream 2,000-bp sequences of the genes were extracted and the cis-acting regulatory elements were scanned in the PlantCARE website (http://bioinformatics.psb.ugent.be/webtools/plantcare/html/) (Lescot et al., 2002).

### Phylogenetic Analysis

FAD protein sequences of prokaryotic algae, eukaryotic algae, bryophytes, pteridophytes, and other plants (Supplementary Table S1) were downloaded from the National Center for Biotechnology Information (NCBI, https://www.ncbi.nlm.nih.gov/)*.* A phylogenetic tree of PfFAD sequences was constructed using the neighbor-joining (NJ) method with MEGA-X (MEGA_X_11.0.1) software with a bootstrap value of 1,000. The online software tool iTOL (https://itol.embl.de/) was used for visualization (Kumar et al., 2018; Letunic and Bork, 2019).

### Gene Expression Analysis

Transcriptome data from different perilla tissues were downloaded from NCBI (accession number: SRP111892) (Zhang et al., 2017), the fragments per kilobase of transcript per million mapped reads (FPKM) values of *FAD* gene families were extracted, and TBtools software was used for clustering and visual analysis (Chen et al., 2020).

qRT-PCR was used to analyze the expression of *FAD* genes. Total RNA was extracted from the plant tissues using a Quick RNA Isolation Kit (TaKaRa, Beijing, China), and cDNA was synthesized from the RNA using a PrimeScript RT Reagent Kit (TaKaRa, China). qRT-PCR reactions were performed using the 2x SYBR Green Pro Taq HS Premix (Accurate, Dongguan, China). Each 10-µl reaction mixture contained 5 µl 2x SYBR Green Pro Taq HS Premix, 0.3 µl of each primer, and 4.4 µl cDNA diluted 20 times. The qPCR cycling conditions were 1 cycle of pre-denaturation at 95°C, 30 s; 40 cycles of 95°C for 5 s and 60°C for 30 s; dissolution at 95°C for 5 s; and 60°C for 1 min. Each experiment was performed four times, and the data were processed by the 2^−ΔΔCT^ method (Livak and Schmittgen, 2001). *AtActin* and *PfActin* were used as endogenous control genes to normalize the semiquantitative RT-PCR analysis of plants. Primers used for qRT-PCR are listed in Supplementary Table S2.

### Gene Cloning and Arabidopsis Transformation

Primers with *Bam*HI and *Sal*I enzyme loci were used for *PfFAD3.1* gene amplification. The amplified CDS of *PfFAD3.1* was digested with the restriction enzymes and inserted into the pCAMBIA2301-KY vector [under the control of the cauliflower mosaic virus (CaMV) 35S promoter] to generate the *35S::PfFAD3.1* construct. The construct was transformed into *Agrobacterium tumefaciens* strain EHA105 and introduced into *A. thaliana* using the floral dip method (Clough and Bent, 1998). The transformed seeds were germinated on half-strength Murashige and Skoog medium with 40 mg/l kanamycin. Seedlings with well-established roots and green leaves were transferred to moistened potting soil and were verified by the PCR method and GUS staining (Supplementary Figure S1). The transgenic T1 lines exhibiting a 3:1 segregation of resistance to 40 mg/l kanamycin were considered the T2 generation, and T2 transgenic lines were finally acquired. Mature seeds of the T2 generation overexpression *PfFAD3.1* Arabidopsis were collected for gene expression and FA content analyses.

### FA Analysis

Dry Arabidopsis seeds (0.15 g), pulverized in liquid nitrogen and FA methyl esters (FAMEs), were prepared according to the method by Li et al. (Li et al., 2015). FAMEs were analyzed using a GC8890 instrument (Agilent, Santa Clara, CA, USA) and a 30 m × 0.25 mm DB-23 column with a nitrogen carrier. The gas chromatography (GC) procedure used the following parameters: split ratio 10:1, initial column temperature held at 100°C for 2 min, then raised to 230°C at the rate of 15°C min^−1^ with the final temperature held for 5 min; injection port temperature 230°C; transfer line temperature 250°C; ion source temperature 230 °C; speed of carrier gas 1.0 ml min^−1^; and injection volume 1 μL. Methyl heptadecanoate was used as an internal standard, and a standard curve method was used as the quantitative approach to identify FAMEs (Yin et al., 2018).

## Results and Analysis

### Identification and Analysis of *FADS* in Perilla

We identified 39 and 58 *PfFAD* candidate genes in the perilla genome using HMM searches and BLAST alignment, respectively. After merging the data, the pfam database was used to predict the conserved domains of the protein encoded by the candidate genes. Thirteen genes lacking the intact FAD domain and two genes with partial FAD domains were identified as pseudogenes and removed. A total of 42 protein-coding genes were confirmed finally, which are located on the other 18 perilla chromosomes except for the 14th and 18th chromosomes (Supplementary Figure S1). The protein sizes range from 178 to 1,111 amino acids, with molecular weights ranging from 20.91 to 126.81 kDa and theoretical isoelectric points ranging from 5.21 to 9.51 (Supplementary Table S3).

**FIGURE 1:**
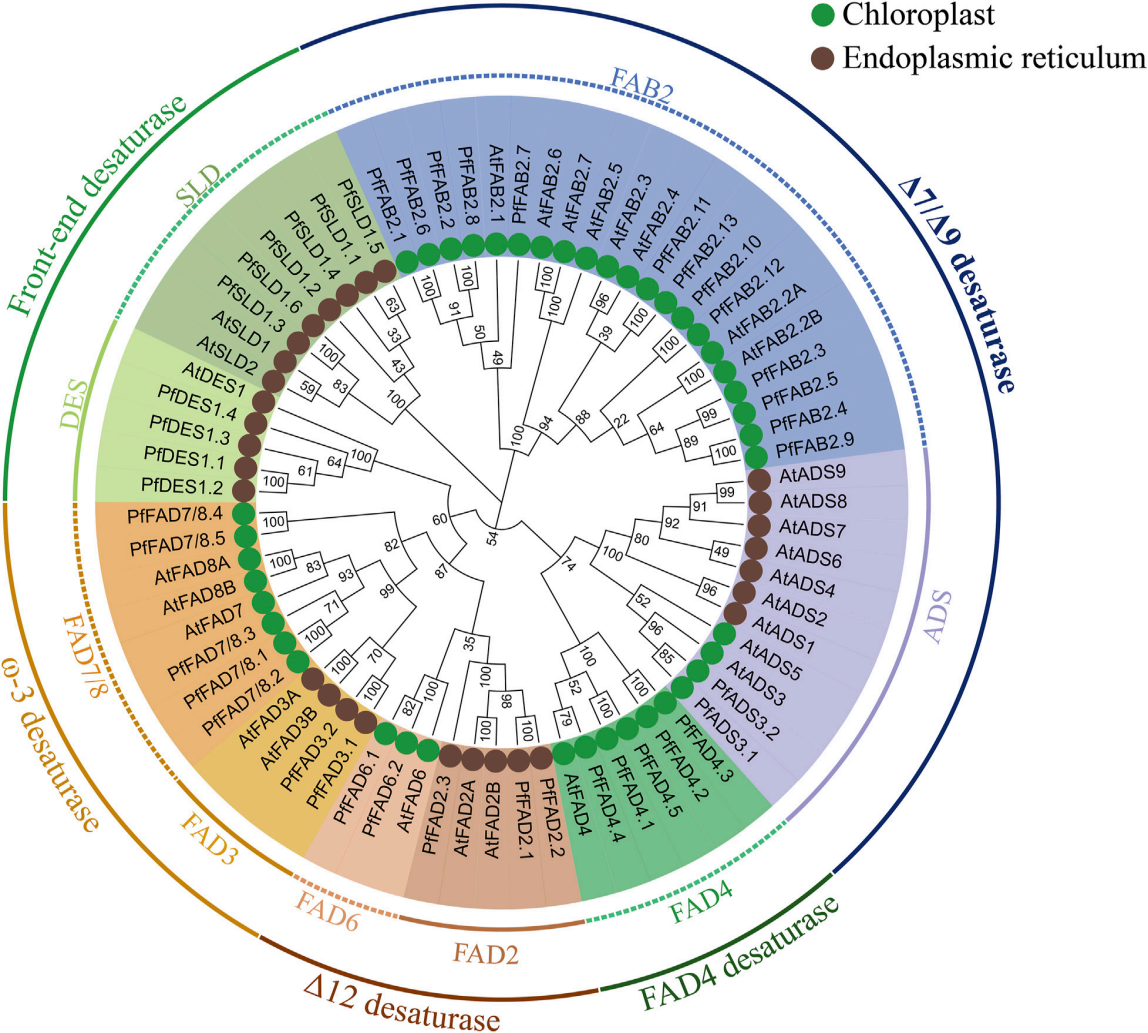
Phylogenetic relationships of *FAD* genes from *perilla* and *Arabidopsis*. The tree was constructed using MEGA X by the neighbor-joining method with 1,000 bootstraps. Subcellular localizations were predicted using ExPASy combined with Cell-PLoc.

**FIGURE 2:**
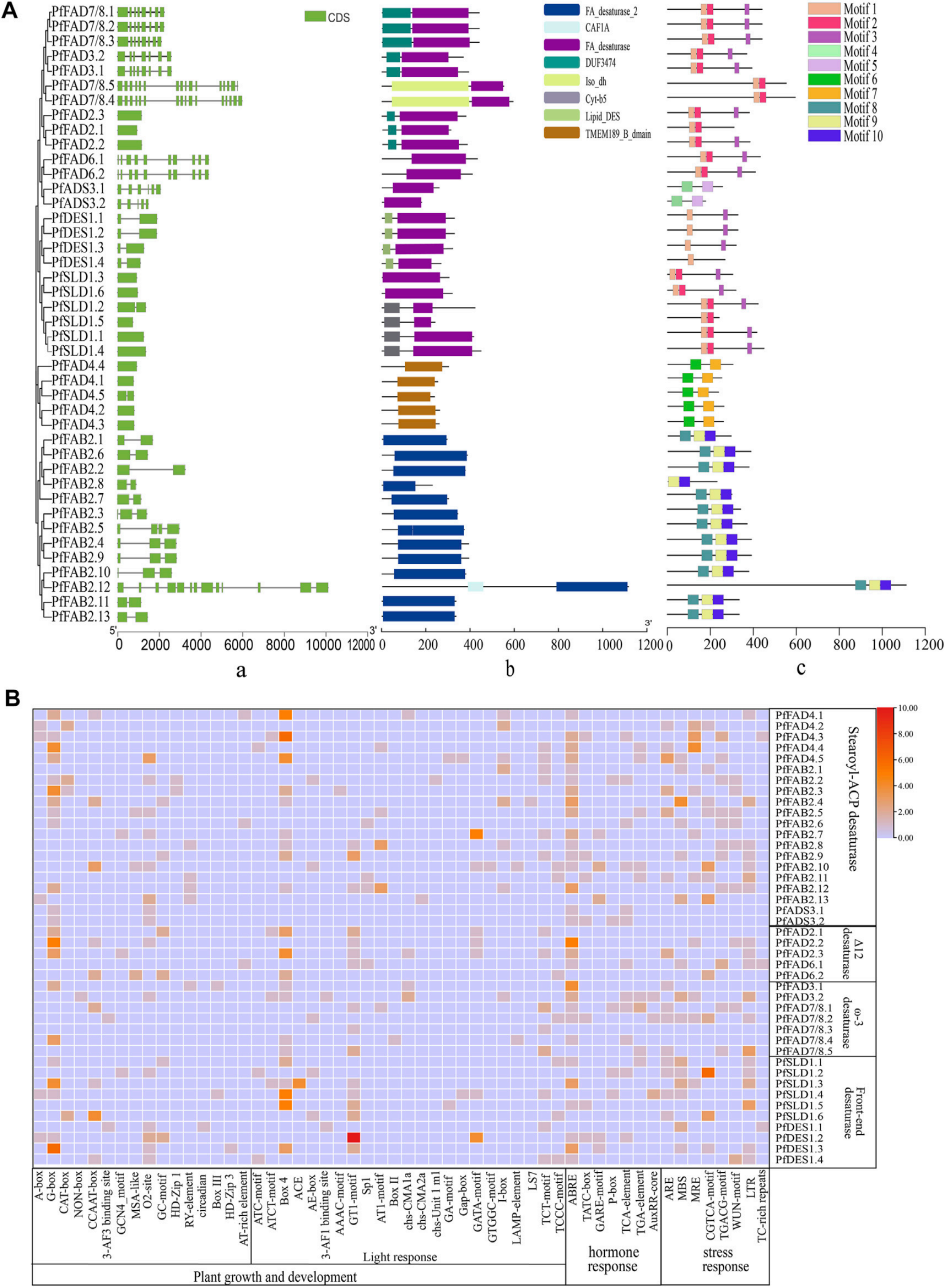
Structure and cis-acting elements of *FADs* in perilla. **(A)** Analysis of gene structures **(i)**, domains **(ii)**, and motifs **(iii)** of the PfFADs. Introns/exons were identified using the GSDS tool. Conserved domains and motifs were analyzed using Pfam and MEME. **(B)** Analysis of the cis-acting elements in the promoters of *PfFADs*. The cis-acting regulatory elements in the upstream 2,000-bp sequences of the genes were identified using the PlantCARE website. Orange and red shading indicates the number of cis-acting elements present; light blue shading means no cis-acting elements were present.

By comparison with the *A. thaliana FAD* family, the *PfFAD* genes were divided into five typical subfamilies based on phylogenetic analysis and sequence characteristics, including Δ7/Δ9 desaturases (Δ7/Δ9 FAD), Δ12 desaturases (Δ12 FAD), ω-3 desaturases (ω-3 FAD), front-end desaturases, and FAD4 desaturases (Figure 1). The Δ7/Δ9 desaturase subfamily contains 15 genes belonging to two types (Kazaz et al., 2020). Thirteen *PfFAB2* genes encode Δ9 desaturases containing an FA_desaturase2 domain (PF03405), and three conserved motifs (8, 9, 10), which were predicted to lack transmembrane domains (TMDs). Two *PfADS3* genes encode Δ7 desaturases with an FA_desaturase domain (PF00487), two motifs (4, 5), and one or two TMDs (Figure 2A and Supplementary Table S3). Δ7/Δ9 desaturases in perilla were predicted to localize in chloroplast. The novel PfFAD4 desaturase subfamily includes five genes, which contain the TMEM189_B_domain (PF10520) and two motifs (6 and 7).

Front-end desaturase, Δ12 desaturase, and ω3 desaturase proteins all contain FA_desaturase as the main domain. The front-end desaturase subfamily includes 10 genes, which encode endoplasmic reticulum-localized proteins and is divided into two clades. Four of these genes (*PfDES*s) encode sphingolipid Δ4 desaturases, which contain two exons and conserved motifs (1 and 3). Both PfDES1.1 and PfDES1.2 lack TMDs, and the remaining two genes encode proteins with five TMDs. Six *PfSLD* genes encode sphingolipid Δ8 desaturases, which contain one conserved motif (1, 2, or 3) but no introns.

The two types of Δ12 desaturase subfamily members contain three conserved motifs (1, 2, and 3). Among them, two *PfFAD6* genes contain 10 exons and encode proteins that only contain the FA_desaturase domain, which were predicted to have a chloroplast signal peptide at the N-termini and three or four TMDs (Xue et al., 2021). Three *PfFAD2* genes lack introns and encode proteins with an FA_desaturase domain and one short DUF3474 domain. PfFAD2 proteins were predicted to contain an endoplasmic reticulum retention signal peptide embedded within Φ-X-X-K-Φ (Φ is a hydrophobic amino acid residue) and three to six TMDs.

ω-3 desaturases are key enzymes for ALA formation (Lee et al., 2016). Here, seven ω-3 desaturase genes were identified in perilla, which were classified into two types (*PfFAD3* and *PfFAD7/8*) consistent with previous classification reports. Two *PfFAD3* genes encode proteins containing an endoplasmic reticulum retention signal (SKKI) at their C-termini and three *PfFAD7/8* genes contain a chloroplast transit peptide at their N-termini*.* Both types contain seven or eight exons and encode proteins with three TMDs, including an FA_desaturase domain and one longer DUF374 domain. Most of these genes contained four motifs (1, 2, and 3). However, there are two special *PfFAD7/8* genes (*PfFAD7/8.4* and *PfFAD7/8.5*) in perilla, which contain 16 exons, more than other *PfFAD7/8* genes in perilla, and other species (Xue et al., 2018). The proteins encoded by these genes contain chloroplast signals but no TMDs, and they also contain an Iso_dh domain in their N-termini in addition to the FA_desaturase domain.

Highly conserved histidine motifs are pivotal for maintaining the catalytic activity of FAD proteins. Based on our predictions, all three histidine boxes were present in the front-end desaturases, Δ12 desaturases, and ω-3 desaturases (Supplementary Table S4). Among the ω-3 desaturases, the histidine-rich motifs HDCGHG, HRTHH, and HVAHH were identified in PfFAD3 and HHDCGH/T(I)AVGHG, HRTHHQN, and HVAHH were identified in PfFAD7/8. The histidine-rich motifs HECG(A)HH, HR(D)RHH, and HVA (IP)HH were identified in Δ12 desaturases. PfFAD4 desaturase subfamily members also contain three histidine boxes: FQG (LD/Y)HH, HA/SWAH, and HAA (TK)HH. However, there are also exceptions. Among front-end desaturases, the histidine-rich motifs HDSGH, HNAHH, and QLEHHL were identified in all SLD1 proteins except PfSLD1.5, and the histidine-rich motifs HELSH, HLEHH, and HNEHH were identified in all DES1 proteins except PfDES1.4. Among Δ7/Δ9 desaturases, PfFAB2 of Δ9 desaturase subfamily members contain two highly conserved histidine-rich motifs, EENRHG and DEKRHE, but lack the third histidine-rich motif. PfADS3 of Δ7 desaturase subfamily members contain the HRHHH and HNNHH motifs but lack the first histidine-rich motif (Kazaz et al., 2020) (Supplementary Table S4).

The cis-regulatory elements in the promoter regions of *PfFAD* family genes were predicted using the online PlantCARE software (Figure 2B). Many light-responsive elements, hormone-responsive elements, and stress-responsive elements were identified in the promoters of *PfFAD* genes. Abscisic acid–responsive elements (ABREs) were widespread in the promoters of 30 *PfFAD* genes, and the ABRE motif was especially enriched in *PfFAD2.1* and *PfFAD3.1.* The GT1-motif and box4 light-responsive elements were widespread in the promoters of *PfFAD* genes. These results suggest that *PfFAD* genes might be involved in stress response and hormonal regulation.

### Expression Analysis of *PfFAD* Family Genes

**FIGURE 3:**
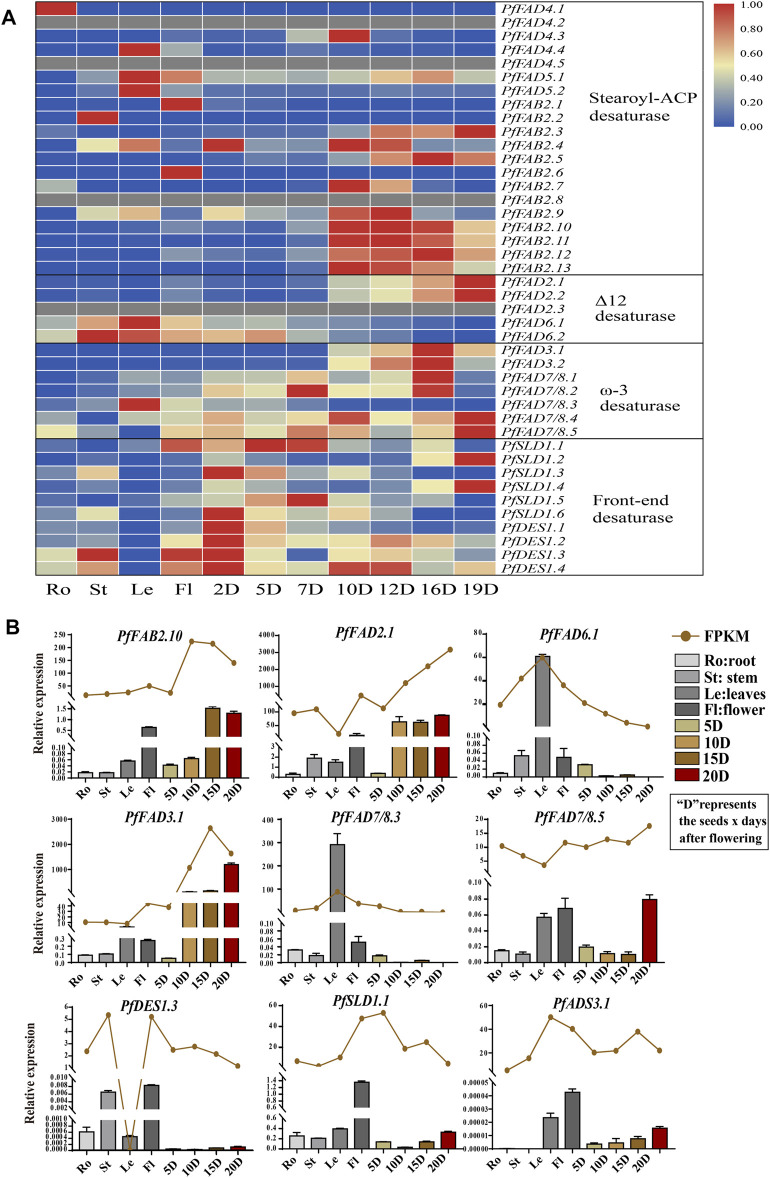
Expression profiles of *FAD* genes in *P. frutescens.* **(A)** Expression patterns of *PfFADs* based on fragments per kilobase of transcript per million mapped reads (FPKM) from perilla. The color gradient blue–white–red indicates low to high levels of gene expression. **(B)** Relative expression levels of *PfFAD* genes determined by qRT-PCR and shown as FPKM. The perilla *Actin* gene was used as an internal control for normalization. Error bars represent SD from at least three biological replicates. Ro, roots; St, stems; Le, leaves; Fl, flowers; 2D–20D, seeds at 2–20 days after flowering.

Based on transcriptome data from different tissues (Figure 3A and Supplementary Table S5), among Δ9 desaturases, most *PfFAB2* genes were highly expressed during the middle and later periods of seed development, except for *PfFAB2.1* and *PfFAB2.6*, which were expressed at very high levels in flowers, and *PfFAB2.2* was highly expressed in stems. Two *PfADS3* genes were highly expressed in leaves, and five *PfFAD4* genes were highly expressed in roots and seeds during the middle stages of development. Among genes in the front-end desaturases, *PfDES1* expression increased during early seed development, and *PfSLD1* genes showed two major expression patterns during early and later seed development. Among genes in the Δ12 FAD subfamily, *PfFAD6.1* and *PfFAD6.2* were mainly expressed in leaves, stems, and flowers, and *PfFAD2.1* and *PfFAD2.2* expressions were increased during later seed development. *PfFAD2.3* was not expressed in all tissues. Among ω-3 FAD subfamily members, *PfFAD3.1* and *PfFAD3.2* were highly expressed during the middle and later stages of seed development, whereas *PfFAD7/8.3* expression increased in leaves. *PfFAD7/8.4* and *PfFAD7/8.5* expression increased during the early and later stages of seed development.

To verify the expression patterns, we examined tissue-specific gene expression patterns by qRT-PCR (Figure 3B). These patterns showed the same tendency as the FPKM. The expression of *PfFAD3.1* and *PfFAD2.1* increased during seed development, which were highly expressed during the later stages of seed development. *PfFAD7/8.3* and *PfFAD6.1* were highly expressed in leaves. *PfFAD7/8.5* was highly expressed in leaves, flower, and the later stage of seed development. *PfDES1.3*, *PfSLD1.1*, and *PfADS3.1* were expressed in flowers, and *PfFAB2.10* was expressed in flowers and during the later stages of seed development. The various expression patterns of *PfFAD* genes imply their functional differentiation.

### Phylogenetic Analysis

**FIGURE 4:**
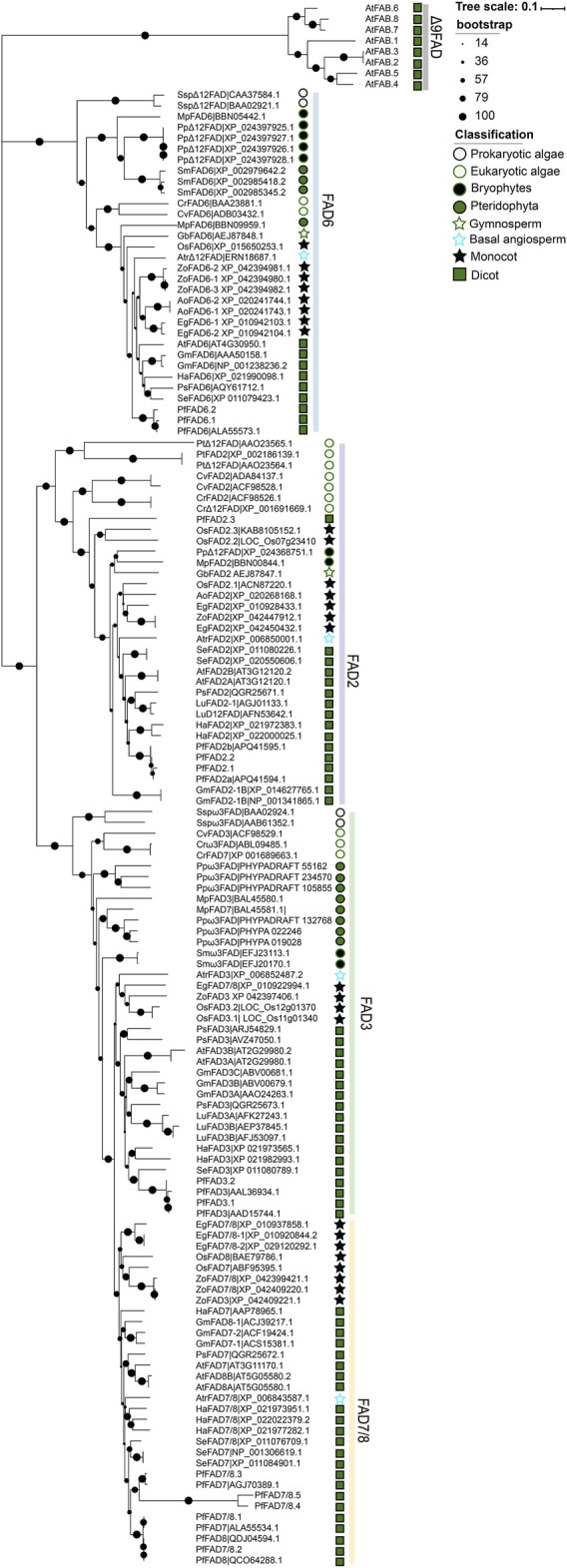
Evolutionary analysis of PUFA desaturases. Δ9 desaturases from *A. thaliana* was used as the outgroup. The tree was constructed using MEGA X by the neighbor-joining method with 1,000 bootstraps. Detailed information on prokaryotic algae, eukaryotic algae, bryophytes, pteridophytes, and monocot and dicot plants is shown in Supplementary Table S1.

To investigate the evolutionary relationships of PUFA desaturase genes in plants, we performed a phylogenetic analysis of Δ12 FAD and ω-3 FAD subfamily members in algae and plants using *A. thaliana* Δ9 desaturases (AtFAB2s) as the outgroup (Figure 4 and Supplementary Table S1). FAD6 of the Δ12 desaturases differentiated earlier than FAD2 and ω-3 desaturases (FAD3 and FAD7/8). The ancestral Δ12 desaturases arose first in prokaryotic algae. Then, they independently differentiated to form Δ12 desaturases in bryophytes and pteridophytes and chloroplast-localized FAD6 in eukaryotic algae and higher plants. The endoplasmic reticulum-localized FAD2 derived from prokaryotic algae Δ12 desaturases. FAD2 independently evolved in eukaryotic algae and lost introns during evolution. ω-3 FAD also arose in prokaryotic algae. From prokaryotic algae to pteridophytes, the differentiation of ω-3 FAD was consistent with the evolution of terrestrial plants. A differentiation event subsequently occurred during the seed plant formation period to form the endoplasmic reticulum-localized FAD3 and the chloroplast-localized FAD7/8 groups in higher plants. Both Δ12 FAD and ω-3 FAD diverged earlier in monocotyledonous plants than dicotyledonous plants. Perilla FAD3 and FAD7/8 appear to be the latest diverged ω-3 FAD in dicotyledons. These findings shed light on the evolution and functional differentiation of FADs.

### Heterologous Overexpression of *PfFAD3.1*

Based on previous reports and our genetic analysis, we speculated that *PfFAD3.1* is a key gene responsible for ALA biosynthesis in perilla seed. To further explore the function of *PfFAD3.1*, we amplified the full-length cDNA sequence and transformed it into *Arabidopsis* plants driven by the CaMV 35S promoter (Supplementary Figure S2) (Lee et al., 2016; Xue et al., 2018). After transgenic screening and identification, six T2-generation transgenic homozygous lines were obtained. Compared with the wild type, the phenotype of leaves and plants, seed size, and thousand seed weight of transgenic lines did not significantly vary (Supplementary Figure S3).

**FIGURE 5:**
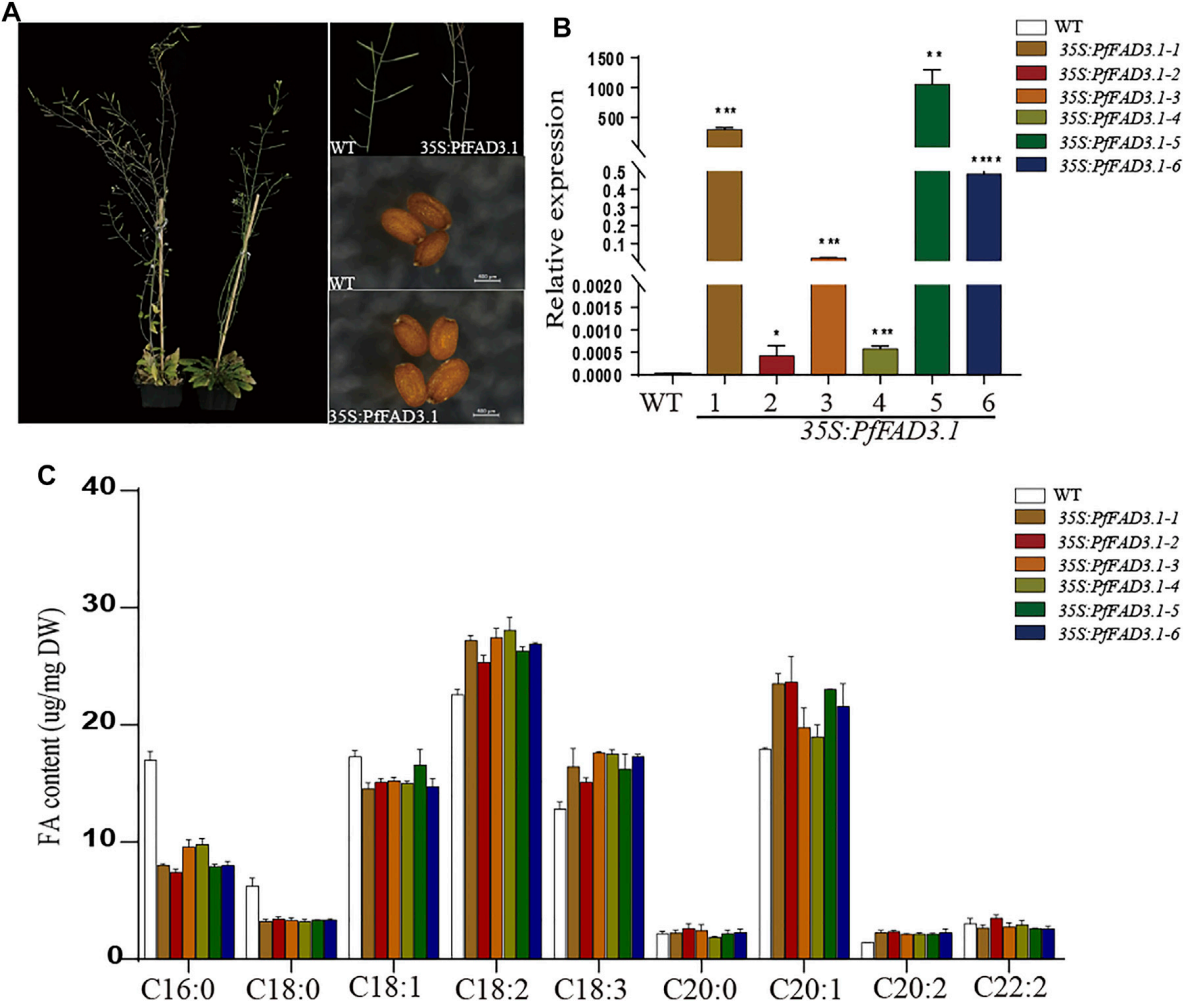
Effect of transgenic *PfFAD3.1* expression on FA composition in *A. thaliana* seeds. **(A)** Transgenic CaMV35S::PfFAD3.1 Arabidopsis plants and seeds. **(B)** The expression of *PfFAD3.1* transgenic 35S::PfFAD3.1 Arabidopsis seeds determined by qRT-PCR. **(C)** FA composition in the seeds of transgenic 35S::PfFAD3.1 Arabidopsis. Error bars represent SD from at least three biological replicates. The asterisks indicate significant differences (*p < 0.05, Student’s t-test).

Then, we analyzed the composition of FAs in mature seeds of these plants using GC (Figure 5C). Compared with the wild type, the ratios of linoleic acid (C18:2) and ALA (C18:3) in transgenic CaMV35S::PfFAD3.1 *Arabidopsis* seeds were increased by 12.27%–24.36% and 17.68%–37.03%, respectively. Moreover, in the transgenic *Arabidopsis* seeds compared to the wild type, the ratios of the other long-chain unsaturated FAs (C20:1 and C20:2) were also higher (6.02%–32.13% and 49.50%–65.35%), and the ratios of palmitic acid (C16:0), stearic acid (C18:0), and oleic acid (C18:1) were decreased by 42.34%–56.29%, 45.58%–48.87%, and 3.97–15.85%, respectively.

**FIGURE 6:**
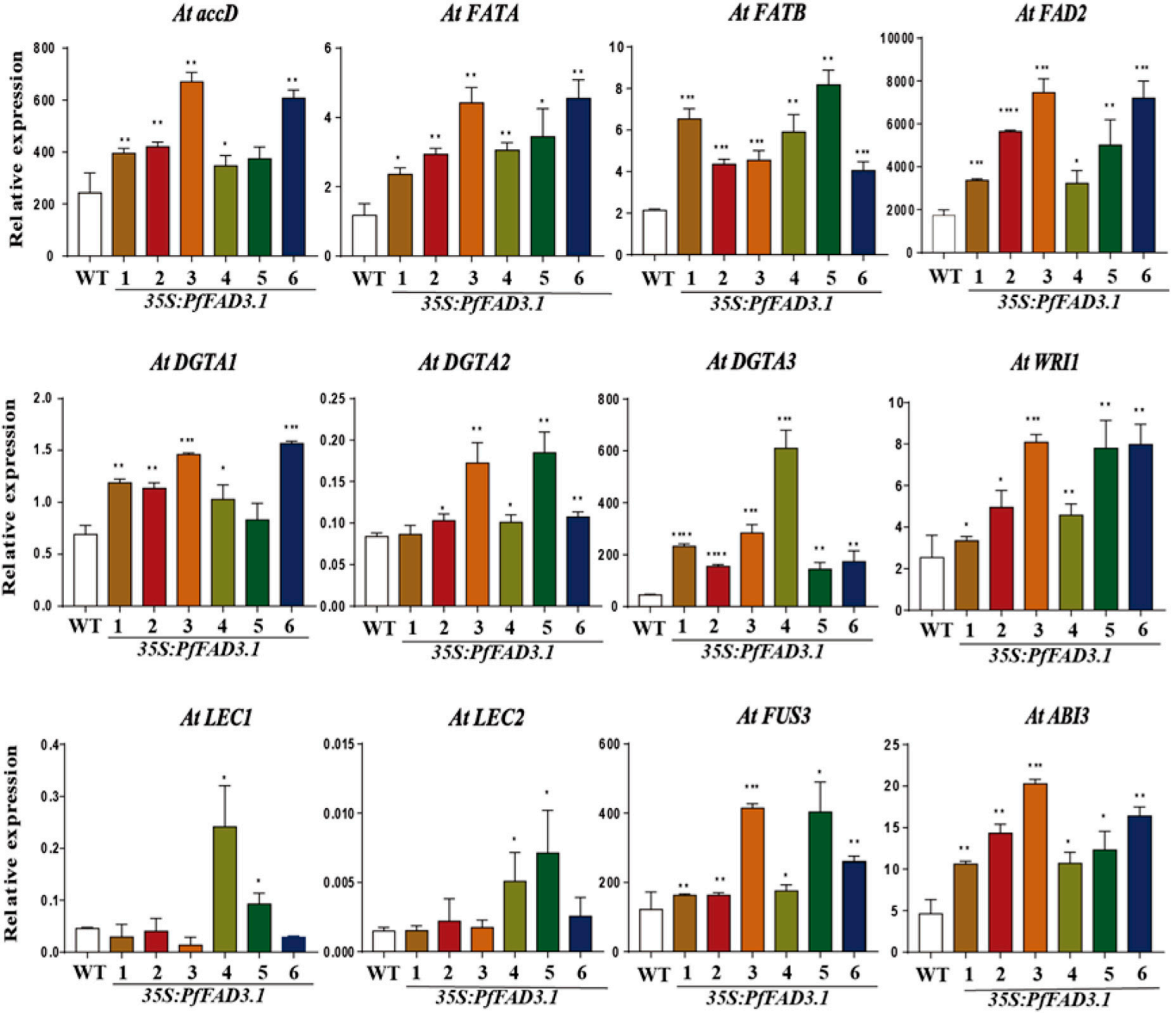
The expression patterns of genes encoding key enzymes and transcription factors involved in α-linolenic acid biosynthesis in 35S::PfFAD3.1 *Arabidopsis* seeds. Error bars represent SD from at least three biological replicates. The asterisks indicate significant differences (*p < 0.05, Student’s t-test).

To investigate the possible role of *PfFAD3.1* in regulating seed lipid biosynthesis, we examined the expression levels of genes involved in lipid biosynthesis and regulation in *Arabidopsis* seeds by qRT-PCR (Figure 6). Genes encoding key enzymes involved in lipid biosynthesis, including the acetyl-CoA carboxylase (ACCase) gene *AtaccD*, fatty acyl-ACP thioesterase (FAT) genes *AtFATA* and *AtFATB*, diacylglycerol acyltransferase (DGAT) genes *AtDGTA1-3*, and fatty acid desaturase 2 gene *AtFAD2*, were upregulated to different degrees in seeds of six transgenic lines. *WRINKLED1* (*WRI1*), *LEAFY COTYLEDON1* (*LEC1*) and *LEC2*, *ABSCISIC ACID INSENSITIVE3* (*ABI3*), and *FUSCA3* (*FUS3*) were reported as positive regulators of seed maturation and seed storage lipid accumulation in *Arabidopsis* (Mendoza et al., 2005; Mu et al., 2008; Elahi et al., 2016). The expression levels of *WRI1*, *ABI3*, and *FUS3* were upregulated in all transgenic *PfFAD3.1 Arabidopsis* lines, and *LEC1* and *LEC2* expressions were upregulated in multiple parts of the transgenic plants.

## Discussion

FADs catalyze PUFA biosynthesis, which are involved in important physiological processes in the plant kingdom (Alonso et al., 2003). In this study, we identified 42 FAD protein-coding genes in the perilla genome. Similar to FADs in other species, the PfFADs were classified into five subfamilies. Members of each subfamily generally had similar exon/intron structures, conserved domain sequences, and similar subcellular localizations. Δ9 desaturases are soluble proteins located in chloroplasts (Kazaz et al., 2020), which were predicted to possess no TMDs in perilla. Δ12 desaturases and ω-3 desaturases included both endoplasmic reticulum-localized and chloroplast-localized genes (Lee et al., 2016). In the perilla genome, two kinds of desaturases were also identified. In previous reports, the endoplasmic reticulum-localized FAD2 and FAD3 were highly expressed in seeds and catalyze ALA accumulation in triacylglycerol biosynthesis in plant seeds. Similarly, *PfFAD3* and *PfFAD2* expressions were increased during seed development in perilla (Rajwade et al., 2014; Xue et al., 2018). FAD6 and FAD7/8 encoded chloroplast-localized proteins that play non-redundant roles in plant responses to low temperature and drought stresses in previous studies (Ángela et al., 2015; Xue et al., 2021). Here, *PfFAD6* and *PfFAD7/8* were highly expressed in perilla leaves. The expression patterns of Δ12 and ω-3 desaturases were in accordance with their proposed functions. Interestingly, *PfFAD7/8.4* and *PfFAD7/8.5* were predicted as special ω-3 desaturases, which possess various gene structures (contained 16 exons and encoded proteins lacking TMD motifs) and expression patterns (upregulated expression in leaves and flowers and in the later stage of seed development) compared with other ω-3 desaturases.

Previous evolutionary analysis of FADs suggested that they share a common origin, and the Δ9 desaturases are the ancestor of membrane desaturase genes (Alonso et al., 2003; Nitta et al., 2005). In the present study, phylogenetic analysis showed that Δ12 and ω-3 desaturases share a common origin. The chloroplast-localized *FAD6* is previous PUFA desaturases, which diverged before *FAD2* and ω-3 desaturase genes. Most *ω-3FAD* genes are present in single copy in algae before evolution during land plant colonization (Xue et al., 2018). In this work, inner differentiation events of ω-3FAD appeared to occur during seed plants formation.

The phylogenetic relationships among PUFA desaturases are strongly associated with their sub-functionalization. Δ9 desaturases, which catalyze the conversion of stearic acid (C18:0) to oleic acid (C18:1), are the most ancestral group of FADs (Kachroo et al., 2007; Zhang et al., 2015). Δ12 desaturases, which differentiated from Δ9 desaturases, catalyze the second desaturase step from oleic acid (C18:1) to linoleic acid (C18:2) (Okuley et al., 1994). ω-3 desaturases, as latter differentiated from Δ12 desaturases, catalyze the desaturase step from linoleic acid (C18:2) to ALA (C18:3) (Shah et al., 1997). Δ9 desaturases are the only desaturases present in most animals and plants, whereas Δ12 and ω-3 desaturases are absent in animal evolutionary lineages.

FAD3 is a primary enzyme catalyzing ALA (C18:3) production in plant seeds. In previous reports, the expression levels of *AtFAD3* are connected with the seed C18:3 accumulation in *Arabidopsis* (Shah et al., 1997). In *Arabidopsis* seeds of overexpression *AtFAD3*, ALA contents decreased by approximately 20% (James and Dooner, 1990; Lemieux et al., 1990). Perilla seeds contain high levels of ALA. In this work, we observed high levels of expression of *PfFAD3.1* during seed development. The *PfFAD3.1* were cloned, which encoded proteins sharing 100% homology to *PfFAD3b* and *PfrFAD3-2* in previous reports (Supplementary Figure S4) (Lee et al., 2016; Xue et al., 2018). *PfFAD3b* was previously cloned and transformed into the budding yeast *Saccharomyces cerevisiae*. However, the transformed yeast produced limited amounts of ALA (1.3% ALA content) (Lee et al., 2016). These results showed that the yeast system does not explain well the function of *PfFAD3*.*1*. Hence, we constructed the 35S::PfFAD3.1 overexpression vector and transformed it into *Arabidopsis*. GC analysis showed that the ratios of ALA were obviously increased in 35S::PfFAD3.1-overexpressing *Arabidopsis* seeds. In a previous report, overexpression of tree peony *PsFAD3* significantly increased (0.6–1.5 times) the ALA content in seeds from five positive transgenic lines. The accumulation of ALA was accompanied with decreased palmitic acid and increased stearic acid and oleic acid (Yin et al., 2018). In the work, the obviously increased ALA contents were along with upregulated linoleic acid (C18:2) and other long-chain PUFA contents and downregulated palmitic acid (C16:0), stearic acid (C18:0), and oleic acid (C18:1) contents in transgenic *Arabidopsis* seeds. The same conclusion showed that *PfFAD3.1* possibly regulated FA metabolic flux, which not only increased ALA biosynthesis and PUFA content but also reduced saturated and monounsaturated FA contents in the heterologous plant system.

Seed maturation in higher plants is associated with the deposition of storage reserves, such as oil accumulation in oilseed plants (Bryant et al., 2019). Several important genes and transcription factors were involved in oil accumulation and seed maturation. ACCase catalyzes the formation of malonyl-CoA, an essential component in FA biosynthesis (Madoka et al., 2002). FAT is a core enzyme in the FA biosynthesis process that determines FA chain length. DGAT catalyzes the covalent addition of a fatty acyl chain to diacylglycerol. In our work, those key genes involved in lipid biosynthesis were expressed at higher levels in transgenic 35S::PfFAD3.1 *Arabidopsis* plants. *WRI1* of the APETALA2/ethylene-responsive (AP2/EREB) family was the key transcription factor regulating seed maturity and FA biosynthesis (Baud et al., 2007). LEC1, an NF-Y family trimeric transcription factor, activates the B3-domain protein LEC2 during early seed development and targets *ABI3* and *FUS3* during the later stages of embryogenesis in *Arabidopsis* (Boulard et al., 2017). In previous reports, *LEC2* and *FUS3* can target *WRI1* to regulate FA biosynthesis (Wang and Perry, 2013; Na et al., 2019). In our work, *WRI1*, *ABI3*, *FUS3*, *LEC1*, and *LEC2* were upregulated in the transgenic seeds. These results suggest that the heterogeneous overexpression of PfFAD3.1 can influence the expression of key biosynthesis genes and transcription factors involved in FA biosynthesis in seed. These results suggest that the heterogeneous overexpression of PfFAD3.1 can influence the expression of key biosynthesis genes and transcription factors involved in FA biosynthesis in seed. However, the detailed molecular mechanisms remain unclean. We will prioritize the study of the mechanism by which *PfFAD3.1* may cause genetic changes related to seed development and will also explore the role of other *PfFADs* in the synthesis of ALA. It is expected to build a foundation for elucidating the functions of perilla FADs and ALA biosynthesis.

## Data Availability

The datasets presented in this study can be found in online repositories. The names of the repository/repositories and accession number(s) can be found in the article/Supplementary Material
