# Supplementary material for: Genome-Wide Analysis of the Fatty Acid Desaturase Gene Family Reveals the Key Role of PfFAD3 in α-Linolenic Acid Biosynthesis in Perilla Seeds
Source: Front Genet. 2021 Nov 24;12:735862. doi: 10.3389/fgene.2021.735862 (PMC8652209; doi:10.3389/fgene.2021.735862)
Supplement: Supplementary file 1 [file DataSheet2.docx]

>PfFAD4.1

MELQQKSSAVIKPLQTQNEESRHVSTWAHRAWFGGGCATMLFSIAKSLILTGRGSRPWLESMLAAMAAYLVADLGAGIYHWAIDNYGSAQTPIFGSQIEGFQGHHQQPWLITKTQLANKLHITAAAVTVAAIPINVLCNEPVLLLFVGVFSACIIFSQQFHAWAHTPKGKLPPLVVALQDAGIILRQAEHAAHHRPPFNSNYCIVSGIWNRALDKSKFFLALEVVVVKVLGHRPRSWNDPSSGWTEISGVN

>PfFAD4.2

MAEGKKNPNSDFSSSQHNRDERCYKATWTHRAWFACGCATVLIALAKSALILAAAPPLTPFTWIQLLLAALLGYVLADLGSGIYHWAIDNYGGAQTPVFGPQIESFLDHHQHPVEITKCETAGILYTVAEVVTTAVTPINILSGDPIFLAFVAVFAGCGMFSLKFHAWAHTPRSKLPPLVAALQDAGVLLRWSEHTKHHRPPYNSDFCTVSGIWNRVLDESNILAAVEAALFRATGVRPRSWSESDPKLGACADVAQNSNE

>PfFAD4.3

MAEIKKNPNSTFSSSQCNVDESWYKSTRTHRAWFACGCATVLIALAKSAFILAAAAPPLTLFIWIQLLLAASLGYVLADLGSGIYHWAIDNYGGDQTPVFGPQIESFLYHHQHPAEITKCETAGILCTVAEVVTVVVTPINILSDHPIFLAFVAVFTGCVMFSLKFHAWAHTPRRKLPPLVTALQDAGVLLKWSEHTKHHRPPFNSHFCTVSGIWNRVLDEFNILAAFEVALFRATGVRPRSWSEPDSGGTEASPNSKE

>PfFAD4.4

MAILPQRHPSLNHARILNHIAPPTHLHSSAAAPPKPRRPDSTTLATPTTTSRPPVLNDPTLQSTWSHRAWLASGCTTVLVSLAKSVAAAAESHIWLEPIIAGLIGYVLADLGSGVYHWGIDNYGGADTPVFGSQIDGFQGHHKWPWTITRRQFANNLHALARATTFVVAPINLVCDDATALGFVAACSGFIMFSQQFHAWAHTTKSRLPAVVAALQDGGVLVSRSQHAAHHRPPYNNNYCIVSGVWNEILDGCKFFEALEMVIFFKLGVRPRSWTEPNSAWTEEPQTTSSLSEHRRKEIVSHQ

>PfFAD4.5

MELQQKSSAVIKPLQTQNEESRHVSTWAHRAWFGGGCATVLFSIAKSLILTGRGSRPWLESMLAAMAAYLVADLGAGIYHWAIDNYGSAQTPIFGSQIEGFQGHHQQPWLITKTQLANKLHITAAAVTVAAIPINVVQQFHSWAHTPKGKLPPLVVALQDAGIILRQAEHAAHHRPPFNSNYCIVSGICNRALDKSKFFAALEVVVVKALGRRPRSWNDPSSGWTEISGVNYSGSS

>PfFAB2.1

MDEFMDEVRALRERTNGLADEYFVVLVGDMITEEALPTYQTGMNSLDGVRDETGASPSPWSTWIRAWSAEENRHGDLLKTYLYLSGRVDMVMIERTLQYLIGSGMAVGWDNNPYLAFVYTSFQERATFVSHGNTARLAKEGGDLLLAGICGTIAADEKRHENAYVKIIEKLLEIDSNETMLAIGNMMRKKIIMPAHLMYDGRDPHLFESFSHVANRLGVYTSNDYADILEFLIKRWKLEKVEGLKGDGRCEQEFVCNLAPRIRKVQERADERAKKMDKQGLKFSWIFNKQVHI

>PfFAB2.2

MQTLTFTTSPLACMLSGGATNLRRPQPIKNKVAPSCRHQTYQPLRPDKVEVFKSLESWASEHVVSLLKPVERCWQPSEFLPDATRPDNDFVDEVRALRERTNGLPDDYFVVLVGDMVTEDALPTYQSALNSLVGVRDETGASPSPWATWIRAWTAEENRHGDLLRSYLYLSGRVDMLMIDRTVQYLIGAGMDHSKWENDPYLGFVFTSFQERATFVSHSNTARLAKEGGDPILAKICGTIAADEKRHENAYVRIIEKLLEIDPNETMVAIGKMLRGKILMPAHLMYDGQDPHLFQHFAHVAHRIGVYTPDDYTGVLESLIGRWRLEKLQGLRDNGKCEQEFVCSLPCRIKTLKVFTNKKAKEGFEGIKFNWIFNKK

>PfFAB2.3

MMRAVIMIMNSNNLCVHGLIFCRVVKTVKKPFSPPHEVHVQVTHSMPQEKIEIFKALEDWAEDNVLIHLKPVEKSWQPQDFLPDPSSDEFHDQVKELRERAREIPDDYFVVLVGDMVTEEALPTYQTMLNTLDGVRDETGASPTSWAIWNRAWTAEENRHGDLLNKYLYLSGRVDMRQIEKTIQYLIGSGMDPQTENNPYLGFIYTSFQERATFLSHGTTARLAREHGDLKLAQICGTIAADEKRHETAYTKISEKLFEIDPDATVLAFADMMRKKISMPARLVYDGRDDNLFDNFSAVAQRLGVYTAKDYADILDHLVSRWNVASLTGLSAEGQRAGICL

>PfFAB2.4

MALKLNAINFQSPKCPSFSLPPAASFRSPKFFMASTLRSGSKEVETAKKPFSPPREVHVQVTHSMPPQKIEIFKSLEDWAEDNILVHLKPVEKCWQPQDFLPDPASDGFHDQVKELRERAKEIPDDYFVVLVGDMITEEALPTYQTMLNTLDGVRDETGASLTPWAVWTRAWTAEENRHGDLLNKYLYLCGRVDMKQIEKTIQYLIGSGMDPRTENSPYLGFIYTSFQERATFVSHGNTARQAREHGDLKLAQICGTIASDEKRHETAYTKIVEKLFEIDPDGTVQSFADMMRKKISMPAHLMYDGRDDNLFDHFSAVAQRLGVYTARDYADILEHLVVRWKVADLTGLSSEGAESSGIRLWIDSENQTARGESTRAGQAGTEDPIQLDIR

>PfFAB2.5

MALKLNAITFQSPKCTSFALPPVATLQSPIFCMASTLGSASKVVKTVKKPFSPPHEVHVQVTHSMPPEKIEIFKALEDWAEDNVLIHLKPVEKSWQPQDFLPDPSSDEFHDQVKELRERAREIPDDYFVVLVGEYGASPTSWAIWNRAWTAEENRHGDLLNKYLYLSGRVDMRQIEKTIQYLIGSGMDPRTENNPYLGFIYTSFQERATFLSHGTTARLAREHGDLKLAQICGTIAADEKRHETAYTKISEKLFEIDPDATVLAFADMMRKKISMPAHLVYDGRDDNLFDNFSAVAQRLGIYTAKDYADILDHLVSRWNVASLTGLSAEGQKAQEYVCELSPRIRRLEERAQARAKQRSRMPFSWIYDREV

>PfFAB2.6

MQSSALFTTTCPVASFPSGGAITLRRSKPVSVVANQPRHPSHHHKISHSMPPEKAEVFKSLEFWASKNILPLLKPVTKCWQPNELLPDPAKPVDEFMDEVRALRERTNRLADEYFVVLVGDMITEEALPTYQTGMNSLDGVRDETGASPSPWSTWIRAWSAEENRHGDLLKTYLYLSGRVDMVMIERTLQYLIGSGMAVGWDNNPYLAFVYTSFQERATFVSHGNTARLAKEGGDPLLAGICGTIAADEKRHENAYVKIIEKLLEIDSNETMLAIGNMMRKKIIMPAHLMYDGRDPHLFESFSHVANRLGVYTSNDYADILEFLIKRWKLEKVEGLKGDGRCEQEFVCNLAPRIRKVQERADERAKKMDKQGLKFSWIFNKQVHI

>PfFAB2.7

MQALAFATPLSCTPRRLPPPSAVSAPLRHHQITHSMPPEKVEVFKSLESWASNHLLPLLKPVDKCWQPNEFLPDPARSFGDFVDEVRALRDRASGLPDEYFVVLVGDMITEDALPTYQTMINTLDGVRDETGASPSPWASWTRAWTAEENRHGDLLRAYLYLSGRVDMLMIERTVQYLIGSGMDPRTENNPYLGYVYTSFQERATFVSHGNMARLAKEGGDSALARICGTIAADEKRHENAYIKIVEKLLEVDPNGSMMAIGDMMRKKITMPAHLMYDGRDPHLFDHFSGRGATAGSIHD

>PfFAB2.8

MSFQERATFVSHSNTARLEKEGGDPILTKICRTIAADEKRHENAYIKIIEKLLEINPNEIMVVIEKMLRGKILIPTHLMYDDRDTHLFQHFTHMAYRIGVYTPDDYTDVLESLIRRWRLEKLQGLKGNDKCEQEFVWSLPRRIKTLKWHVCTFIDASFFNARIMESGMASATEQERQPPTFLPVQRAKPGSFDLSGLGVPEEGQRMINELMLMYENNVEEGNKNNLI

>PfFAB2.9

MALKLNAINFQSPKCPSFSLPPAASFRSPKFFMASTLRSGSKEVETAKKPFGPPREVHVQVTHSMPPQKIEIFKSIEDWAEDNILVHLKPVEKCWQPQDFLPDPASDGFHDQVKELRERAKEIPDDYFVVLVGDMITEEALPTYQTMLNTLDGVRDETGASLTPWAVWTRAWTAEENRHGDLLNKYLYLCGRVDMKQIEKTIQYLIGSGMDPRTENSPYLGFIYTSFQERATFVSHGNTARQAREHGDLKLAQICGTIASDEKRHETAYTKIVEKLFEIDPDGTVQSFADMMRKKISMPAHLMYDGRDDNLFDHFSAVAQRLGVYTARDYADILEHLVVRWKVADLTGLSSERAESSGIRLWIDSENQTARGESTRAGQAGTEDPIQLDIR

>PfFAB2.10

MLLFRSLSTDKSGTCLRESSFVSSRDAGKGKKPFSSPREVRVQVTHPLPPEKREIFDSLQDWAEENILVLLKPVEKCWQPNDFLPDPSSEGFEEQVRELRKRTKEIPDDYFVVLVGDMITEEALPTYQTMLNTLDSVRDETGASLTPWAIWTRAWTAEENRHGDLLNKYLYLSGRVDMRQIEKTIQYLIGSGMDPGTDKNPYLGFIYTSFQERATFVSHGNTARLAKEHGDLKLAQICGIIAADEKRHETAYTKIVEKLFEIDPDGTVLALADMMRKKISMPAHLMYDGADDNLFEHFSSVAQRLGVYTAKDYADILEFLVARWEVEKLTGLSGEGRKAQDYVCGLPPRIRRLEERAQARVKQAAPVPFSWIYGREVKL

>PfFAB2.11

MPPEKIEIFNSLQDWAENNILVLLKDVEKSWQPTDFLPDSASEGFHEQVKELRERCKEIPDDYFVVLIGDMITEEALPTYQTMINTLDGVRDETGASPTPWAVWTRAWTAEENRHGDLLNKYLYLSGRVDMRQVEKTIQYLIGSGMDPRTENNPYLGFIYTSFQERATFISHGNTARHAKQHGDIKLAQICGTIAADEKRHETAYTKIVEKLFEVDPDGTVLCLADMMRKKITMPAHLMYDGREDSLFDNFSSVAQRLAVYTAKDYADILEFLVAQWEVEKLTGLSGEGRKAQEYVCGLPPRIRRLEERAQGKAKGKEAVRVPFSWIFGREVML

>PfFAB2.12

MENVEKLESSSASLNHVIACLMEESDLPLSILVDEIFEKVKDRIGNGENVTRATVKSSVLMIGQRSCYGLASADADLLEDEAEVALWCWETRDLKLIPKLERVSVKVRRTCRRKIQERITAVTAMVSALEKSEDHPNYRHDLVKASEKLGKVLNEADIRLLMENMSQKNGAEITPLQEKEQKRLRSETEKEEKRREKEEYEMQKQQKRQQEEAEKDQKRKSKEEAELKKQLALQKQASLMEQFLKRKKSASSQNESPSNKAITSISSPDLVEKESETVTANMDSVLVGNAEVEAEAIWKSHLNSWHCIGHSIRSKRNVHWSLRQKPKTELVKELKLTINSEITCDEDQVEEKNVDGLIDPNIDGRQSQISTDRPLPDCQKRIRSMQLLQFDKSHRPAFYGVWPRKSQVIGARHPFAKDQDIDYEIDSDEEWEEEEPGESLSDCEKDEEDESTEEQQKVDDEDESEDEFFVPDGYLSENEGVQTDEMESDELDHQEASNLPNSKVQVQTEEFCTLLRQQKYLINMTEHALKKNQPLIILNLMHEKXQVQTEEFCTLLRQQKYLNNMTEHALKKNQPLIILNLLHEKTTLLPAEELTGSEKIEKMCLQALCIRSFPDFPEAEISIHKDEVDGDLEASSKKSSPTPPATAAAISDSDLPQIISIINSCPHGIGKIAGSLHNMFPAFSKSQLRNKVREISEFSENRWQVKKEILSKFGLSISPVEMALKLCVTPSKMPSFPGSQLRSHRVSMASTIHSPSVDAGKGKKPFSSPREVRVQVTHPLPPEKREIFDSLQDWAEENILVLLKPVEKCWQPNDFLPDPSSEGFEEQVRELRKRTKEIPDDYFVVLVGDMITEEALPTYQTMLNTLDSVRDETGASLTPWAIWTRAWTAEENRHGDLLNKYLYLSGRVDMRQIEKTIQYLIGSGMDPGTDKNPYLGFIYTSFQERATFVSHGNTARLAKEHGDLKLAQICGIIAADEKRHETAYTKIVEKLFEIDPDGTVLALADMMRKKISMPAHLMYDGADDNLFEHFSSVAQRLGVYTAKDYADILEFLVARWEVEKLTGLSGEGRKAQDYVCGLPPRIRRLEERAQTRVKQAAPVPFSWIYGREVKL

>PfFAB2.13

MPPEKIEIFNSLQDWAENNILVLLKDVEKSWQPTDFLPDSASEGFHEQVKELRERCKEIPDDYFVVLIGDMITEEALPTYQTMINTLDGVRDETGASPTPWAVWTRAWTAEENRHGDLLNKYLYLSGRVDMRQVEKTIQYLIGSGMDPRTENNPYLGFIYTSFQERATFISHGNTARHAKQHGDIKLAQICGTIAADEKRHETAYTKIVEKLFEVDPDGTVLCLADMMRKKITMPAHLMYDGREDSLFDNFSSVAQRLAVYTAKDYADILEFLVAQWKVEKLTGLSGEGRKAQEYVCGLPPRIRRLEERAQGKAKGKEAVRVPFSWIFGREVML

>PfADS3.1

MKRVRPIVCAASIPVPGDGKDSNFGRILLSDVVVKRRRNLFRGRNWSALDIAAGNPIDWVSTHRHHHQFCDSEKDPHSPLEGFWFSHMSWMFDTKTIVERCGEPNNVGDLEKQPFYKFIQKTYIFHSMALGALLYAMGGFPYIVWGMRGPFDSGPKGVRIVWVYHITWLVNSACHVWGKQEWNTGDLSRNNWWVAMLAFGEGWHNNHHAFEYSARHGLEWWQIDMTWYAIKALEAIGLATDVKLPTPAQKQKMAFAK

>PfADS3.2

MLDLQGNPIDWVSTHRHHHQFCDSEKDPHSPLEGFWFSHMSWMFDTKTIVERCGEPNNVGDLEKQPFYKFIEKTYIFHSVALGALLYSMGGFPYIVWGMRGPFDSGPKGVRIVWVYHITWLVNSACHVWGKQAWNTGDLSRNNWWVAMLAFGEGWHNNHHAFEYSARHGLEWWQIDMT

>PfFAD2.1

MGAGGRMSVPPEGKKAKSVVERVPFTKPPFTLGEIKKAIPPHCFKRSIPRSFSYVLYDLVIASLFYYVATNYFHQLPYPLSYVAWPLYMICQGCILTGVWVIAHECGHHAFSDYQWLDDTVGLVLHSFLLVPYFSWKYSHRRHHSNTGSLERDEVFVPKVKSALGSSAKYLNNPPGRILTLIVQFTLGWPLYLMFNVSGRPYDRFACHFDPKSPIYSDRERAQIFLSDVGILAMLYGLYRLTLAKGLAWVLCVYGVPLLVVNGFLVLITYLQHTHASLPHYDSSEWDWLRGALSTVDRDYGVLNTVFQ

>PfFAD2.2

MGAGGRMSVPPEGKKAKSVVERVPFTKPPFTLGEIKKAIPPHCFKRSIPRSFSYVLYDLVIASLFYYVATNYFHQLPYPLSYVAWPLYMICQGCILTGVWVIAHECGHHAFSDYQWLDDTVGLVLHSFLLVPYFSWKYSHRRHHSNTGSLERDEVFVPKVKSALGSSAKYLNNPPGRILTLIVQFTLGWPLYLMFNVSGRPYDRFACHFDPKSPIYSDRERAQIFLSDVGILAMLYGLYRLTVAKGLAWVLCVYGVPLLVVNGFLVLITYLQHTHASLPHYDSSEWDWLRGALSTVDRDYGVLNTVFHNITDTHVAHHLFSTMPHYHAMEATKAIKPILGEYYQFDGTPVAKAVWREAKECVYVEPDEGDKNKGVFWYNNKL

>PfFAD2.3

MGAESRALPPPAGLKSGRAHHTKPPFTLAEIKKSIPPHCFRRSILRSFSYVAHDVAAASLLYYAAANYIHRLPRPLPHLAWPAYWFAQGCVFTGLWGIAHDCGHHAFSDIQAVDDGVGLILHSFLLVPYFSFKFSHRRHHSNTNSLDRDEVYVPKMKSEIHRSIKYLNNPIGRVIMLLIQLSIGFPLYLIFNASGRRYGRIANHFDPYSPIYKEGERAAVIISDAAILVVIHALCYLAGAKGVGWLLAVYGGPMVACNAFVGAITYLQHTHPSLPRYDSSEWEWLKATLSTVDRDYGRVLNEVFHHTADTHVVHHLFPTIPHYHAVEATAAVKPVLGEHYLFDEMPVVKALWRAAKECLYVEREEGGENKGVFWFNYKI

>PfFAD6.1

MACRLAHSGFLFLGPQKRPNEGNRIFPQSSTSSGTCLLKWESLPRRLSKQKQCLISIRKREIVKAVAVTVAPSPAADNAEYRQQLCHEYGFRQIGEPLPDNVFEIDDAKSWKSVLISITSYALGIFMIAKAPWYLLPLAWAWTGTAITGFFVIGHDCAHKSFSRNKLVEDIVGTLAFMPLIYPYEPWRFKHDRHHAKTNMLEEDTAWLPVNPEDLESSILRKALIYAYGPFRPWMSIAHWLRMHFDVKNFRPNEVNRVKISLACVFAFMAIGWPLMIYKTGIMGWIKFWLMPWLGYHFWMSTFTMVHHTAPHIPFKSSDEWNAAKAQLNGTVHCDYPSWIEVLCHDINVHIPHHISPRIPSYNLRAAHKSLQENWGKYMNEASWNWRLMKTILTVCHVYDKEKNYAPFDEIVPEESQPITFLKQVMPDYA

>PfFAD6.2

MACRLAHSGFLFLGPQKRPNEGNRIFPQSSTSSVAPSPAADNAEYRQQLCHEYGFRQIGEPLPDNVTLKDILDTLPKKVFEIDDAKSWKSVLISITSYALGIFMIAKAPWYLLPLAWAWTGTAITGFFVIGHDCAHKSFSRNKLVEDIVGTLAFMPLIYPYEPWRFKHDRHHAKTNMLEEDTAWLPVNPEDLESSILRKALIYAYGPFRPWMSIAHWLRMHFDVKNFRPNEVNRVKISLACVFAFMAIGWPLMIYKTGIMGWIKFWLMPWLGYHFWMSTFTMVHHTAPHIPFKSSDEWNAAKAQLNGTVHCDYPSWIEVLCHDINVHIPHHISPRIPSYNLRAAHKSLQENWGKYMNEASWNWRLMKTILTVCHVYDKEKNYAPFDEIVPEESQPITFLKQVMPDYA

>PfFAD3.1

MAVSSGARLSKSGADGEVFDGQQQYEGIGKRAADKFDPAAPPPFKIADIRAAIPAHCWVKNPWRSLSYVVWDVAAVFALLAAAVYINSWAFWPVYWIAQGTMFWALFVLGHDCGHGSFSDNTTLNNVVGHVLHSSILVPYHGWRISHRTHHQNHGHVEKDESWVPLPENLYKKLDFSTKFLRYKIPFPMFAYPLYLWYRSPGKTGSHFNPYSDLFKPNERGLIVTSTMCWAAMGVFLLYASTIVGPNMMFKLYGVPYLIFVMWLDTVTYLHHHGYDKKLPWYRSKEWSYLRGGLTTVDQDYGFFNKIHHDIGTHVIHHLFPQIPHYHLVEATREAKRVLGNYYREPRKSGPVPLHLIPALLKSLGRDHYVSDNGDIVYYQTDDELFPSKKI

>PfFAD3.2

MAVSSGARLSKSGADGEVFDGQQQYEGIGKRAADKFDPAAPPPFKIADIRAAIPAHCWVKSPWRSLSYVVWDVAAVFALLAAAVYINSWAFWPVYWIAQGTMFWALFVLGHDCGHGSFSDNTTLNNVVGHVLHSSILVPYHGWRISHRTHHQNHGHVEKDESWVPWYRSPGKTGSHFNPYSDLFKPNERGLIVTSTMCWAAMGVFLLYASTIVGPNMMFKLYGVPYLIFVMWLDTVTYLHHHGYDKKLPWYRSKEWSYLRGGLTTVDQDYGFFNKIHHDIGTHVIHHLFPQIPHYHLVEASCSTSYSTREAKRVLGNYYREPRKSGPVPLHLIPALLKSLGRDHYVSDNGDIVYYQTDDELFPSKKI

>PfFAD7/8.1

MASFVISECGLKPLPRIYPKPRAAQPLSSSNLRFSRTNQRFNSSFCSSSGINKERNWALRVSAPLRIQPVEEENRAINGGEEFDPAAPPPFKLSDIKAAIPKHCWVKDPWRSVSYVVRDVVAVFGMAAAAAYFNNWLVWPLYWFAQSTLFWALFVLGHDCGHGSFSNNPKLNSVFGHLLHSSILVPYHGWRISHRTHHQNHGHVENDESWHPLPEKIYNSLDNNTKMLRFTLPFPMLAYPFYLWSRSPGKKGSHFHPESDLFVPNERKDVITSTVCWTAMAALLVGLSFVIGPLQLLKLYGIPYLGFVAWLDLVTYLHHHGHEDKLPWYRGKEWSYLRGGLTTLDRDYGLINNIHHDIGTHVIHHLFPQIPHYHLIEATEAAKGVLGKYYREPKKSGPLPLHLLGELLRSMKKDHYVSDTGDIVYYQTDPQLNGGRKS

>PfFAD7/8.2

MASFVISECGLKPLPRIYPKPRAAQPLSSSNLRFSRTNQRFNSSFCSSTGIIKERNWALRVSAPLRIQPVEEENRAINGGEEFDPAAPPPFKLSDIKAAIPKHCWVKDPWRSVSYVVRDVVAVFGMAAAAAYFNNWLVWPLYWFAQSTLFWALFVLGHDCGHGSFSNNPKLNSVFGHLLHSSILVPYHGWRISHRTHHQNHGHVENDESWHPLPEKIYNSLDNNTKMLRFTLPFPMLAYPFYLWSRSPGKKGSHFHPESDLFVPNERKDVITSTVCWTAMAALLVGLSFVIGPLQLLKLYGVPYLGFVAWLDLVTYLHHHGHEDKLPWYRGKEWSYLRGGLTTLDRDYGLINNIHHDIGTHVIHHLFPQIPHYHLIEATEAAKGVLGKYYREPKKSGPLPLHLLGDLLRSMKKDHYVSDTGDIVYYQTDPQLNGGRKS

>PfFAD7/8.3

MASWVLSECGLRPLPRIYPKPRTDQYVSNSNPSRLRVSRTGFSSDSSFSLVGRERNWGLKVSAPLRFQEVEEESEERGSVVVNGVDEFDPGAPPPFKLSDIRAAIPKHCWVKDPWRSMSYVVRDVVVVFGLAAGAAYFNNWAVWPLYWFAQSTMFWALFVLGHDCGHGSFSNDPKLNSVAGHLLHSSILVPYHGWRISHRTHHQNHGHVENDESWHPIPEKIYKTLDFATKKLRFTLPFPMLAYPFYLWGRSPGKKGSHFHPDSDLFVPNERKDVITSTVCWTAMLAILAGLSFVMGPVQLLKLYGIPYIGFVAWLDLVTYLHHHGHDEKLPWYRGKEWSYLRGGLTTLDRDYGWINNIHHDIGTHVIHHLFPQIPHYHLIEATAAAKPVLGKYYKEPKKSGPFPFYLLGVLQKSMKKDHYVSDTGDIVYYQTDPELN

>PfFAD7/8.4

MAASLQLNCKPFTPLLLHSRSASKRAPKFSTVRCSAASPSKSYTITLLPGDGIGPEVISVAKNALKLVASLEGFAFKFTEVPMGGAALDLTGVPLPEETLSIAKGSDAVLLGAIGGYKWDNNEKHLKPETGLLQLRAGLKVFANLRPATVLPQLVDASTLKKDVAEGVDLMVVRELTGGIYFGKPRGFGKNENGEEIGFNTEVYTTHEIDRIARVAFETARKRGGKLCSVDKANVLEASMLWRKRVTALASEYPDVELSHMYVDNAAMQLVRNPKQFDTIVTNNIFGDILSDEASMITGSIGMLPSASLGESGPGLFEPIHGSAPDIAGQDKANPLATVLSAAMLLKYGLGEEKAARRIEAAVLDTLDRGYRTGDIHSAGQKLVGCREMGEQVLKSIDSKVATAVGHGSFSNDPKLNSVAGHLLHSSILAPYHGWRISHRTHNQNHGHVENDESWHPIPEKIYRTLDFATKKLRFTLPFPMLAYPFYLLGRSPGKKGSHFHPDSDLFVPIERKDVITSTVCWTAMVAGLSFVTGPAHLSEFHASLYNLKMELGLQGFVAWLDLVTYLHHHGHDEKLPWYRGMELPETGAHDT

>PfFAD7/8.5

MAASLQLNCKPFTPLLLHSKSASKRAPKFSTVHCSAASPSKSYTITLLPGDGIGPEVISVAKNALKLVASLEGFAFKFTEVPVGGAALDLTGVPLPEETLSIAKGSDAVLLGAIGGYKWDNNEKHLKPETGLLQLRAGLKVFANLRPATVLPQLVDASTLKKDVAEGVDLMVVRELTGGIYFGKPRGFGKNENGEEIGFNTEVYTTHEIDRIARVAFETARKRGGKLCSVDKANVLEASMLWRKRVTALASEYPDVELSHMYVDNAAMQLVRNPKQFDTIVTNNIFGDILSDEASMITGSIGMLPSASLGESGPGLFEPIHGSAPDIAGQDKANPLATVLSAAMLLKYGLGEEKAARRIEAAVLDTLDRGYRTGDIHSAGQSGEEMGEQVLKSIDSKVTIAVGHGSFSNDPKLNSVAGHLLHSSILVPYHGWRISHRTHHQNHGHVENDESWHPIPEKIYRTLDFATKKLRFTLPFPMLAYPFYLWGRSPGKKGSHFHPDSDLFVPNERKDVITSTVCWTAMVAGLSFVTGPAQLLKTLWHTLYWICGMA

>PfSLD1.1

MADQHKKYITSDELKKHNKPHDLWLSIQGKIYNVTDWSKIHPGGPIPLMNLAGQDVTDAFIAFHPASAWKFLDNFFTGYYLQDFEVSEVSRDYRNLSAKFTKSGLFEKKGHGTILSLCFVTLLLSACFYGVLRSDSFFIHMLSGGLLGFTWMQIAYLGHDSGHYNIMTSRGFNKFVQILTGNCLTGISIAWWKWTHNAHHVSCNSLDYDPDLQHLPVLAVSSKLFQSLTSRFYLRELTFDPLSRFFVSYQHFTFYPVMCVARVNLYLQTLLLLFSKRKVPDRALNILGIAVFWTWFPLLVSCLPNWSERVLFVLASFCVCAIQHVQFCLNHFAANVYVGAPKGNDWFEKQTSGTIDIACSPKMDWFFGGLQFQLEHHLFPRLPRCNLRKISPVVQELCKKHNLPYTESLLRRGE

>PfSLD1.2

MAAETKKYITSDELKKHNKREDLWLSIQGKVYNVTDWAKQHPGGEVPLMNMAGEDVTDAFIAFHPGSAWKHLDRFFTGYHLQDFHVSEMSRDYRNLANHIAKSGLFENKGHGTIYYLCLVSVLLAACFYGVLRCDDFSIHMLSGAVLGFVWMQVAYLGHDSGHYCMMMNPRFNKVAQILTGNCLTGISIAWWKWTHNAHHIACNSLDYDPDLQHLPMLAVSTSLFKSLTSQFYGRKRIRLGRQILHQLPASHLLPCNVRCQSQSLSSNILAALLPYLTGLRESSSCSRASASVQFSTFSSRSTISPPMSTLGRPNRSEWLEKQTKGTIDISCPSWMDWFFGGLQFQLEHHLFPRLPRCQLRKISPIVEELCKKHNLPYRSLSFFEANKWTLRTLRAAALEARDFTVVPKNLLWEAVNTHG

>PfSLD1.3

MGVMWIQSGWMGHDSGHYQIMLTPNVNRFVQILSGNVLAGISIAWWKRNHNAHHIAVNSLDHDPDLQHMPFFAVSSKLFNSITSFYYGRKMQFDKASRFLISNQHWTFYPVMCFARLNLFAQSFMLLLSKTPVPNRGLELLGLLVFWIWYPLLVSCLPSWSERVLFVAASFVVTSIQHVQFCLNHFSSSVYVGKPKGNDWFEKQTNGSLDISCPSWMDWFHGGLQFQIEHHLFPRLPRGQLRSISPFVRELCKKHGLPYNCATFLGANVLTVRTLRDAAMQARDFTKPLPKNLVWEAVNTHG

>PfSLD1.4

MAAETKKYITSDELKKHNKREDLWLSIQGKVYNVTDWAKQHPGGEVPLMNMAGEDVTDAFIAFHPGSAWKHLDRFFTGYHLQDFHVSEMSRDYRNLANHIAKSGLFENKGHGTIYYLCLVSVLLAACFYGVLRCDDFSIHMLSGAVLGFVWMQVAYLGHDSGHYCMMMNPRFNKVAQILTGNCLTGISIAWWKWTHNAHHIACNSLDYDPDLQHLPMLAVSTSLFKSLTSQFYGRKLEFDSVARFFISYQHLTYYPVMCVARVNLYLQTFLLLLSNRKVPDRALNILGIMVFWTWFPLLVASLPNWTERVLFVLASFCVCSIQHIQFTLNHFAANVYIGPPKSSEWLEKQTKGTIDISCPSWMDWFFGGLQFQLEHHLFPRLPRCQLRKISPIVEELCKKHNLPYRSLSFFEANKWTLRTLRAAALEARDFTVVPKNLLWEAVNTHG

>PfSLD1.5

MADQHKKYITSDELKKHTKPHDLWLSIQGKVYNVTDWSKIHPGGPIPLMNLAGQDVTDAFIAFHPASAWKFLNNFFTGYYLEDFEVSEVSRDYRNLSAKFTKSGLFEKKGHGTVLSLCFVTLLLSACFYGVLRSDSFFIHMLSGGLLGFTWMQIAYLGHDSGHYNIMTSRGFNKFVQILTGNCLTGISIAWWKWTHNAHHVSCNSLDYDPDLQHLARSRGFFEALSISNIALLPQRTHV

>PfSLD1.6

MIIICKKTISXCGALMGVMWIQSGWMGHDSGHYQIMLTPNVNRFVQILSGNVLAGISIAWWKRNHNAHHIAVNSLDHDPDLQHMPFFAVSSKLFNSITSFYYGRKMQFDKASRFLISNQHWTFYPVMCFARLNLFAQSFMLLLSKTPVPNRGLELLGLLVFWIWYPLLVSCLPSWSERVLFVAASFVVTSIQHVQFCLNHFSSSVYVGKPKGNDWFEKQTNGSLDISCPSWMDWFHGGLQFQIEHHLFPRLPRGQLRSISPFVRELCKKHGLPYNCATFLGANVLTVRTLRDAAMQARDFTKPLPKNLVWEAVNTHG

>PfDES1.1

MGFEGEKGVMAADFFWSYTDEPHASRRRQILSQYPQIKQLFGPDPFAFLKVAAVVLLQLWTAAFLNNASWLQILTVAYFFGSFLNHNLFLAIHELSHNLAFSTPVYNRWLGIFANLPIGVPMSVTFQKYHLEHHRYQGVDGVDMDVPSLTEAKVVRNTVSKSIWVVLQLFFYALRPLFLKPKPPGIWEFINLTIQVSLDATIVHFWGWKSFGYLILSTFVGGGMHPMAGHFISEHYVFNPDQETYSYYGPLNLMTWSVGYHNEHHDFPRIPGSKLHKVRDIAPEFYQNLESYQSWSQVIYMYVMDRTVGPFSRMKRNISSSDKKSE

>PfDES1.2

MGFEGEKGVMATDFFWSYTDEPHASRRRQILSQYPQIKQLFGPDPFAFLKVAAVVLLQLWTAAFLNNASWLQILTVAYFFGSFLNHNLFLAIHELSHNLAFSTPVYNRWLGIFANLPIGVPMSVTFQKYHLEHHRYQGVDGVDMDVPSLTEAKVVRNTVSKSIWVVLQLFFYALRPLFLKPKPPGIWEFINLTIQVSLDATIVHFWGWKSFGYLILSTFVGGGMHPMAGHFISEHYVFNPDQETYSYYGPLNLMTWSVGYHNEHHDFPRIPGSKLHKVRDIAPEFYQNLESYQSWSQVIYMYVMDRTVGPFSRMKRNISSSDKKSE

>PfDES1.3

MATDFFWSYTDEPHASRRRIILSKYPQIRQLFGPDPFAFLKIAAIVSIQLWTATFLYNASWLKILIVAYFFGSFLNNNLFLAIHELSHNLAFSTPVYNRWLGIFANLPIGVPMSVTFQKYHLEHHRYQGVDGIDMDIPSLTEAYLVTNAFTKSIWVIMQLFFYALRPLFLKPKPPGLWEFANLIAQLALDVAIVYFWGWKAFGYLILSTFVGGGMHPMAGHFISEHYVFNSEQETYSYYGPLNLMTWNVGFHNEHHDFPRIPGSKLHKVKEIAPEYYDNLDCYRSWSQVIYMYVMDRTVGPFSRMKRSLSVKSLNKSE

>PfDES1.4

MGYQGKESGEEEGVMATDFFWSYTDEPHASRRRIILSKYPQIRQLFGPDPFAFLKIAAIVSIQLWTATFLYNASWLKILIVAYFFGSFLNNNLFLAIHELSHNLAFSTPVYNRWLGIFANLPIGVPMSVTFQKYHLEHHRYQGVDGIDMDIPSLTEAYLVTNAFTKSIWVIMQLFFYALRPLFLKPKPPGLWEFANLIAQLALDVAVVYFWGWKAFGYLISVYICWRWNAPHGRPLYLRTLCFQLGTRDLFLLRSSESNDMERRIP
