# Supplementary material for: Genome-Wide Analysis of the Fatty Acid Desaturase Gene Family Reveals the Key Role of PfFAD3 in α-Linolenic Acid Biosynthesis in Perilla Seeds
Source: Front Genet. 2021 Nov 24;12:735862. doi: 10.3389/fgene.2021.735862 (PMC8652209; doi:10.3389/fgene.2021.735862)
Supplement: Supplementary file 2 [file DataSheet1.docx]

**SUPPORTING INFORMATION**

**TABLE |S1 The information of FAD in other species**

| **Group** | **Organism name** | **Protein name** | **Accession** |
| --- | --- | --- | --- |
| Prokaryotic algae | *Synechocystis sp. PCC* | SspΔ12FAD | BAA02921.1 |
|  |  | SspΔ12FAD | CAA37584.1 |
|  |  | Sspω3FAD | AAB61352.1 |
|  |  | Sspω3FAD | BAA02924.1 |
| Eukaryotic algae | *Phaeodactylum tricornutum* | PtΔ12FAD | AAO23565.1 |
|  |  | PtΔ12FAD | AAO23564.1 |
|  |  | PtFAD2 | XP_002186139.1 |
|  | *Chlorella vulgaris* | CvΔ12FAD | ACF98528.1 |
|  |  | CvFAD6 | ADB03432.1 |
|  |  | CvFAD3 | ACF98529.1 |
|  |  | CvFAD2 | ADA84137.1 |
|  | *Chlamydomonas reinhardtii* | CrΔ12FAD | XP_001691669.1 |
|  |  | CrFAD2 | ACF98526.1 |
|  |  | CrFAD7 | XP_001689663.1 |
|  |  | CrFAD6 | BAA23881.1 |
|  |  | Crω3FAD | ABL09485.1 |
| Bryophytes | *Physcomitrium patens* | PpΔ12FAD | XP_024368751.1 |
|  |  | PpΔ12FAD | XP_024397925.1 |
|  |  | PpΔ12FAD | XP_024397927.1 |
|  |  | PpΔ12FAD | XP_024397928.1 |
|  |  | PpΔ12FAD | XP_024397926.1 |
|  |  | Ppω3FAD | PHYPA_019028 |
|  |  | Ppω3FAD | PHYPA_022246 |
|  |  | Ppω3FAD | PHYPADRAFT_132768 |
|  |  | Ppω3FAD | PHYPADRAFT_105855 |
|  |  | Ppω3FAD | PHYPADRAFT_234570 |
|  |  | Ppω3FAD | PHYPADRAFT_55162 |
|  | *Marchantia polymorpha* | MpFAD2 | BBN00844.1 |
|  |  | MpFAD6 | BBN09959.1 |
|  |  | MpFAD6 | BBN05442.1 |
|  |  | MpFAD7 | BAL45581.1 |
|  |  | MpFAD3 | BAL45580.1 |
| Pteridophyta | *Selaginella moellendorffii* | SmFAD6 | XP_002985345.2 |
|  |  | SmFAD6 | XP_002985418.2 |
|  |  | SmFAD6 | XP_002979642.2 |
|  |  | Smω3FAD | EFJ20170.1 |
|  |  | Smω3FAD | EFJ23113.1 |
| Basal angiosperm | *Amborella trichopoda* | AtrFAD2 | XP_006850001.1 |
|  |  | AtrD12FAD | ERN18687.1 |
|  |  | AtrFAD7/8 | XP_006843587.1 |
|  |  | AtrFAD3 | XP_006852487.2 |
| Gymnosperm | *Ginkgo biloba* | GbFAD6 | AEJ87848.1 |
|  |  | GbFAD2 | AEJ87847.1 |
| Monocot | *Oryza sativa* | OsFAD2.1 | ACN87220.1 |
|  |  | OsFAD2.2 | LOC_Os07g23410 |
|  |  | OsFAD2.3 | KAB8105152.1 |
|  |  | OsFAD6 | XP_015650253.1 |
|  |  | OsFAD3.1 | LOC_Os11g01340 |
|  |  | OsFAD3.2 | LOC_Os12g01370 |
|  |  | OsFAD7 | ABF95395.1 |
|  |  | OsFAD8 | BAE79786.1 |
|  | *Asparagus officinalis* | AoFAD6-1 | XP_020241743.1 |
|  |  | AoFAD6-2 | XP_020241744.1 |
|  |  | AoFAD2 | XP_020268168.1 |
|  | *Elaeis guineensis* | EgFAD6-2 | XP_010942104.1 |
|  |  | EgFAD6-1 | XP_010942103.1 |
|  |  | EgFAD7/8 | XP_010922994.1 |
|  |  | EgFAD7/8 | XP_010937858.1 |
|  |  | EgFAD7/8-2 | XP_029120292.1 |
|  |  | EgFAD7/8-1 | XP_010920844.2 |
|  |  | EgFAD2 | XP_010928433.1 |
|  |  | EgFAD2 | XP_042450432.1 |
|  | *Zingiber officinale* | ZoFAD7/8 | XP_042399421.1 |
|  |  | ZoFAD3 | XP_042409221.1 |
|  |  | ZoFAD7/8 | XP_042409220.1 |
|  |  | ZoFAD3 | XP_042397406.1 |
|  |  | ZoFAD6-3 | XP_042394982.1 |
|  |  | ZoFAD6-2 | XP_042394981.1 |
|  |  | ZoFAD6-1 | XP_042394980.1 |
|  |  | ZoFAD2 | XP_042447912.1 |
| Dicot | *Glycine max* | GmFAD2-1B | NP_001341865.1 |
|  |  | GmFAD2-1B | XP_014627765.1 |
|  |  | GmFAD6 | NP_001238236.2 |
|  |  | GmFAD6 | AAA50158.1 |
|  |  | GmFAD3A | AAO24263.1 |
|  |  | GmFAD3B | ABV00679.1 |
|  |  | GmFAD3C | ABV00681.1 |
|  |  | GmFAD7-1 | ACS15381.1 |
|  |  | GmFAD7-2 | ACF19424.1 |
|  |  | GmFAD8-1 | ACJ39217.1 |
|  | *Linum usitatissimum* | LuFAD2-1 | AGJ01133.1 |
|  |  | LuFAD3B | AFJ53097.1 |
|  |  | LuFAD3A | AFK27243.1 |
|  |  | LuFAD3B | AEP37845.1 |
|  | *Sesamum indicum* | SeFAD2 | XP_020550606.1 |
|  |  | SeFAD2 | XP_011080226.1 |
|  |  | SeFAD6 | XP_011079423.1 |
|  |  | SeFAD7 | XP_011084901.1 |
|  |  | SeFAD7/8 | XP_011076709.1 |
|  |  | SeFAD7 | NP_001306619.1 |
|  |  | SeFAD3 | XP_011080789.1 |
|  | *Helianthus annuus* | HaFAD6 | XP_021990098.1 |
|  |  | HaFAD2 | XP_022000025.1 |
|  |  | HaFAD2 | XP_021972383.1 |
|  |  | HaFAD7 | AAP78965.1 |
|  |  | HaFAD3 | XP_021982993.1 |
|  |  | HaFAD7/8 | XP_021977282.1 |
|  |  | HaFAD7/8 | XP_021973951.1 |
|  |  | HaFAD3 | XP_021973565.1 |
|  |  | HaFAD7/8 | XP_022022379.2 |
|  | *Paeonia suffruticosa* | PsFAD3 | AVZ47050.1 |
|  |  | PsFAD3 | ARJ54829.1 |
|  |  | PsFAD3 | QGR25673.1 |
|  |  | PsFAD7 | QGR25672.1 |
|  |  | PsFAD6 | AQY61712.1 |
|  |  | PsFAD2 | QGR25671.1 |
|  | *Perilla frutescens* | PfFAD3 | AAL36934.1 |
|  |  | PfFAD7 | AGJ70389.1 |
|  |  | PfFAD7 | ALA55534.1 |
|  |  | PfFAD8 | QDJ04594.1 |
|  |  | PfFAD8 | QCO64288.1 |
|  |  | PfFAD2b | APQ41595.1 |
|  |  | PfFAD2a | APQ41594.1 |
|  | *Arabidopsis thaliana* | AtFAB2.1 | AT1G43800.1 |
|  |  | AtFAB2.2A | AT2G43710.1 |
|  |  | AtFAB2.2B | AT2G43710.2 |
|  |  | AtFAB2.3 | AT3G02630.1 |
|  |  | AtFAB2.4 | AT5G16240.1 |
|  |  | AtFAB2.5 | AT5G16230.1 |
|  |  | AtFAB2.6 | AT3G02610.1 |
|  |  | AtFAB2.7 | AT3G02620.1 |
|  |  | AtADS1 | AT1G06080.1 |
|  |  | AtADS2 | AT2G31360.1 |
|  |  | AtADS3/AtFAD5 | AT3G15850.1 |
|  |  | AtADS5 | AT3G15870.1 |
|  |  | AtADS4 | AT1G06350.1 |
|  |  | AtADS6 | AT1G06360.1 |
|  |  | AtADS7 | AT1G06100.1 |
|  |  | AtADS8 | AT1G06090.1 |
|  |  | AtADS9 | AT1G06120.1 |
|  |  | AtFAD2A | AT3G12120.1 |
|  |  | AtFAD2B | AT3G12120.2 |
|  |  | AtFAD6 | AT4G30950.1 |
|  |  | AtFAD4 | AT4G27030.1 |
|  |  | AtFAD3A | AT2G29980.1 |
|  |  | AtFAD3B | AT2G29980.2 |
|  |  | AtFAD7 | AT3G11170.1 |
|  |  | AtFAD8A | AT5G05580.1 |
|  |  | AtFAD8B | AT5G05580.2 |
|  |  | AtDES1 | AT4G04930.1 |
|  |  | AtSLD1 | AT3G61580.1 |
|  |  | AtSLD2 | AT2G46210.1 |

Note: The FAD proteins from various algae and plants were downloaded from NCBI

**TABLE| S2. Primers used in this study.**

| **Gene** | **Accession** | **Primer name** | **Primer sequence (5ʹ–3ʹ)** | **Application** |
| --- | --- | --- | --- | --- |
| *PfFAD3.1* | MZ747492 | *PfFAD3.1-F* | ATGGCCGTTTCTTCCGGT | PCR of full-length cDNA |
|  |  | *PfFAD3.1-R* | CCTAAATCTTTTTGGAAGGAAAGAG | PCR of full-length cDNA |
| *PfFAD3.1* | MZ747492 | *2301-35S-F* | GACGCACAATCCCACTATCC | PCR identification |
|  |  | *PfFAD3.1-R* | CCTAAATCTTTTTGGAAGGAAAGAG | PCR identification |
| *PfActin* | AB002819 | *PfActin-F* | CTCAACCCCAAGGCAAACAG | Reference gene |
|  |  | *PfActin-R* | ATCACGACCAGCAAGATCCAA | Reference gene |
| *PfFAD3.1* | MZ747492 | *PfFAD3.1-RT-F* | CGCAGCAAGGAATGGAGTTAT | qRT-PCR |
|  |  | *PfFAD3.1-RT-R* | GGTAATGTGGGATCTGAGGGA | qRT-PCR |
| *PfFAD7/8.3* | MZ747483 | *PfFAD7/8.3-RT-F* | AGTGTTGCTGGTCACCTGCTT | qRT-PCR |
|  |  | *PfFAD7/8.3-RT-R* | ATCGCTGTCTGGATGGAAATG | qRT-PCR |
| *PfFAD7/8.5* | MZ747502 | *PfFAD7/8.5-RT-F* | TCGCCGTCCAAGAGCTACAC | qRT-PCR |
|  |  | *PfFAD7/8.5-RT-R* | GCAAGGGCACTCCAGTCAAAT | qRT-PCR |
| *PfFAD2.1* | MZ747489 | *PfFAD2.1-RT-F* | CCGGTTCGCATGCCACTT | qRT-PCR |
|  |  | *PfFAD2.1-RT-R* | CCCGTTCACCACGAGCAA | qRT-PCR |
| *PfFAD6.1* | MZ747487 | *PfFAD6.1-RT-F* | CATCAGAAAGCGTGAAATCGT | qRT-PCR |
|  |  | *PfFAD6.1-RT-R* | CCAAGCTAGAGGAAGCAGGT | qRT-PCR |
| *PfDES1.3* | MZ747490 | *PfDES1.3-RT-F* | CACCACCGTTATCAAGGC | qRT-PCR |
|  |  | *PfDES1.3-RT-R* | AGCAACATCAAGGGCAAG | qRT-PCR |
| *PfSLD1.1* | MZ747475 | *PfSLD1.1-RT-F* | GGACTCACAACGCTCACCA | qRT-PCR |
|  |  | *PfSLD1.1-RT-R* | TCTCGCAACGCACATTACA | qRT-PCR |
| *PfADS3.1* | MZ747466 | *PfADS3.1-RT-F* | TGCGAGGACCATTTGATT | qRT-PCR |
|  |  | *PfADS3.1-RT-R* | GCCACCCACCAGTTATTT | qRT-PCR |
| *PfFAB2.10* | MZ747493 | *PfFAB2.10-RT-F* | TTCATCTACACCTCGTTCC | qRT-PCR |
|  |  | *PfFAB2.10-RT-R* | ATCAAGTGAGCTGGCATT | qRT-PCR |
| *AtActin2* | AT3G18780 | *AtActin2-F* | GGTAACATTGTGCTCAGTGGTG | Reference gene |
|  |  | *AtActin2-R* | CTCGGCCTTGGAGATCCACATC | Reference gene |
| *AtaccD* | ATCG00500 | *AtaccD-F* | GTTCAAACAGGTACAGGTCAAC | qRT-PCR |
|  |  | *AtaccD-R* | AAACTTCCTTCTTGCATTCGTG | qRT-PCR |
| *AtDGAT3* | AT1G48300 | *AtDGAT3-F* | GACTGATTCAGTTAGGACACCA | qRT-PCR |
|  |  | *AtDGAT3-R* | AATATGAGACAGAACCGAGTCC | qRT-PCR |
| *AtDGAT2* | AT3G51520 | *AtDGAT2-F* | AAATATGGTCGTAAGCTCGCTA | qRT-PCR |
|  |  | *AtDGAT2-R* | CATAACCAAAGACATAGGCACG | qRT-PCR |
| *AtDGAT1* | AT2G19450 | *AtDGAT1-F* | TTCACCGGATTCATGGGATTTA | qRT-PCR |
|  |  | *AtDGAT1-R* | GAAGAAGCAGTAGAACATGCAG | qRT-PCR |
| *AtFATA* | AT3G25110 | *AtFATA-F* | GCTGTTGTATCTGCTGATCAAG | qRT-PCR |
|  |  | *AtFATA-R* | CATCAGTCGAAAAACCAACACT | qRT-PCR |
| *AtFATB* | AT1G08510 | *AtFATB-F* | CGAGGGGAAATAGAGCCTTATT | qRT-PCR |
|  |  | *AtFATB-R* | AACATAGTCAGCAGTCTTGTCA | qRT-PCR |
| *AtWRI1* | AT3G54320 | *AtWRI1-F* | CTTGTACCTCGGCACCTATAAT | qRT-PCR |
|  |  | *AtWRI1-R* | AATGTCGAAATTAGTAACCGCG | qRT-PCR |
| *AtLEC1* | AT1G21970 | *AtLEC1-F* | CAAGAACAATGGTATCGTGGTC | qRT-PCR |
|  |  | *AtLEC1-R* | ACTCGGAGACACATTCTTGAAT | qRT-PCR |
| *AtLEC2* | AT1G28300 | *AtLEC2-F* | AGGAAGAGAAAATGAGTCGAGG | qRT-PCR |
|  |  | *AtLEC2-R* | CCACCACTCAAAGTCGTTAAAG | qRT-PCR |
| *AtABI3* | AT3G24650 | *AtABI3-F* | CTGTTTCTCACCTTCAACATGG | qRT-PCR |
|  |  | *AtABI3-R* | ATAGTTTGGAGCAGGCATGTAT | qRT-PCR |
| *AtFUS3* | AT3G26790 | *AtFUS3-F* | TTTTGTGAATGCTCATGGTCTG | qRT-PCR |
|  |  | *AtFUS3-R* | ACGTCTACTTCTTCTTCTTCCG | qRT-PCR |
| *AtFAD2* | AT3G12120 | *AtFAD2-F* | CGTCTTGATCACTTACTTGCAG | qRT-PCR |
|  |  | *AtFAD2-R* | ACCTTGTTCAAGATTCCGTAGT | qRT-PCR |

**TABLE | S3. Basic information about FAD genes in *P. frutescens***

| **Gene** | **Accession** | **Position** | **Protein length (aa)** | **Molecular weight (kDa)** | **PI** | **GRAVY** | **Transmembrane zone** | **Exon number** | **Subcellular localization** | **Strand** |
| --- | --- | --- | --- | --- | --- | --- | --- | --- | --- | --- |
| *PfFAD4.1* | MZ747474 | chr19:10524286-10525041 | 251 | 27.56 | 9.14 | 0.133 | 3 | 1 | C | - |
| *PfFAD4.2* | MZ747488 | chr06:61798931-61799716 | 261 | 28.42 | 6.40 | 0.085 | 3 | 1 | C | + |
| *PfFAD4.3* | MZ747496 | chr04:00150653-00151432 | 259 | 28.53 | 6.85 | 0.166 | 4 | 1 | C | + |
| *PfFAD4.4* | MZ747503 | chr04:65514490-65515401 | 303 | 33.23 | 8.65 | -0.117 | 0 | 1 | C | + |
| *PfFAD4.5* | MZ747507 | chr07:22377405-22378175 | 236 | 25.73 | 9.51 | -0.045 | 2 | 2 | C | + |
| *PfFAB2.1* | MZ747468 | chr02:28564756-28566428 | 293 | 33.53 | 5.61 | -0.363 | 0 | 2 | C | - |
| *PfFAB2.2* | MZ747470 | chr05:64747678-64750912 | 376 | 42.78 | 6.84 | -0.375 | 0 | 2 | C | + |
| *PfFAB2.3* | MZ747477 | chr04:62471624-62472880 | 341 | 38.86 | 5.34 | -0.35 | 0 | 3 | C | - |
| *PfFAB2.4* | MZ747479 | chr13:17877302-17880112 | 391 | 44.24 | 5.39 | -0.49 | 0 | 3 | C | + |
| *PfFAB2.5* | MZ747482 | chr06:05036618-05039570 | 371 | 42.34 | 6.15 | -0.43 | 0 | 4 | C | - |
| *PfFAB2.6* | MZ747484 | chr20:25594303-25595735 | 385 | 43.73 | 7.2 | -0.382 | 0 | 2 | C | - |
| *PfFAB2.7* | MZ747485 | chr20:29601609-29602947 | 300 | 33.58 | 5.77 | -0.36 | 0 | 2 | C | - |
| *PfFAB2.8* | MZ747486 | chr11:57487256-57489842 | 227 | 26.43 | 6.19 | -0.466 | 0 | 2 | C | + |
| *PfFAB2.9* | MZ747491 | chr10:44602425-44605244 | 391 | 44.31 | 5.47 | -0.498 | 0 | 3 | C | + |
| *PfFAB2.10* | MZ747493 | chr06:63586129-63588710 | 379 | 43.22 | 5.68 | -0.455 | 0 | 3 | C | - |
| *PfFAB2.11* | MZ747497 | chr17:00390759-00391870 | 334 | 38.25 | 5.21 | -0.452 | 0 | 2 | C | - |
| *PfFAB2.12* | MZ747505 | chr11:02111217-02121223 | 1111 | 126.81 | 5.25 | -0.668 | 0 | 13 | C | + |
| *PfFAB2.13* | MZ747506 | chr15:00242548-00243975 | 334 | 38.25 | 5.36 | -0.453 | 0 | 2 | C | + |
| *PfADS3.1* | MZ747466 | chr09:37067475-37069213 | 257 | 29.64 | 9.14 | -0.319 | 1 | 6 | C | - |
| *PfADS3.2* | MZ747467 | chr03:46310003-46311463 | 178 | 20.91 | 6.20 | -0.395 | 2 | 5 | C | - |
| *PfFAD2.1* | MZ747489 | chr12:56933298-56934223 | 308 | 35.12 | 8.99 | 0.117 | 6 | 1 | ER | - |
| *PfFAD2.2* | MZ747499 | chr11:05592060-05593208 | 382 | 43.65 | 8.64 | -0.018 | 6 | 1 | ER | + |
| *PfFAD2.3* | MZ747500 | chr11:05575254-05576393 | 379 | 42.74 | 8.86 | -0.008 | 3 | 1 | ER | + |
| *PfFAD6.1* | MZ747487 | chr01:69490649-69495019 | 430 | 49.91 | 8.9 | -0.184 | 3 | 10 | C | + |
| *PfFAD6.2* | MZ747504 | chr05:26977876-26982207 | 407 | 47.24 | 8.08 | -0.188 | 4 | 10 | C | - |
| *PfFAD3.1* | MZ747492 | chr12:04645208-04647776 | 391 | 44.93 | 8.93 | -0.202 | 3 | 8 | ER | - |
| *PfFAD3.2* | MZ747469 | chr11:54194712-54197265 | 367 | 41.78 | 8.48 | -0.211 | 3 | 7 | ER | + |
| *PfFAD7/8.1* | MZ747471 | chr04:47415627-47417845 | 438 | 50.03 | 9.13 | -0.304 | 3 | 8 | C | - |
| *PfFAD7/8.2* | MZ747480 | chr06:49937118-49939326 | 438 | 50.01 | 9.13 | -0.286 | 3 | 8 | C | - |
| *PfFAD7/8.3* | MZ747483 | chr15:29789304-29791384 | 438 | 50.18 | 8.63 | -0.296 | 3 | 8 | C | + |
| *PfFAD7/8.4* | MZ747495 | chr09:59833558-59839253 | 592 | 64.43 | 6.93 | -0.152 | 0 | 16 | C | - |
| *PfFAD7/8.5* | MZ747502 | chr03:71866967-71872514 | 550 | 59.67 | 7.34 | -0.12 | 0 | 16 | C | + |
| *PfSLD1.1* | MZ747475 | chr20:37120998-37122241 | 414 | 47.67 | 8.89 | 0.032 | 6 | 1 | ER | + |
| *PfSLD1.2* | MZ747476 | chr12:35417611-35418431 | 420 | 47.84 | 9.07 | -0.201 | 2 | 2 | ER | + |
| *PfSLD1.3* | MZ747478 | chr08:60731497-60732405 | 302 | 34.75 | 9.48 | 0.013 | 0 | 1 | ER | + |
| *PfSLD1.4* | MZ747481 | chr11:24427893-24429236 | 447 | 51.59 | 8.61 | -0.012 | 5 | 1 | ER | + |
| *PfSLD1.5* | MZ747498 | chr02:34170013-34170731 | 239 | 27.15 | 8.3 | -0.1 | 2 | 1 | ER | - |
| *PfSLD1.6* | MZ747501 | chr16:50833429-50834514 | 317 | 36.33 | 9.49 | 0.078 | 3 | 1 | ER | + |
| *PfDES1.1* | MZ747472 | chr07:11773221-11775103 | 326 | 37.72 | 7.94 | -0.055 | 0 | 2 | ER | - |
| *PfDES1.2* | MZ747473 | chr19:01853800-01855666 | 326 | 37.75 | 7.94 | -0.063 | 0 | 2 | ER | + |
| *PfDES1.3* | MZ747490 | chr02:61723372-61724610 | 318 | 37.05 | 8.72 | 0.061 | 5 | 2 | ER | - |
| *PfDES1.4* | MZ747494 | chr05:48893443-48894079 | 266 | 30.97 | 8.77 | 0.155 | 5 | 2 | ER | + |

Note: In the Subcellular localization column, C refers to chloroplast; ER refers to endoplasmic reticulum.

**TABLE | S4. Conserved histidine-rich boxes in fatty acid desaturases in *P.* *frutescens***

| **Subfamily** | **Gene** | **Accession** | **His-box Ⅰ** | **His-box Ⅱ** | **His-box Ⅲ** |
| --- | --- | --- | --- | --- | --- |
| FAD4 desaturase | *PfFAD4.1* | MZ747474 | FQGHH | HAWAH | HAAHH |
|  | *PfFAD4.2* | MZ747488 | FLDHH | HAWAH | HTKHH |
|  | *PfFAD4.3* | MZ747496 | FLYHH | HAWAH | HTKHH |
|  | *PfFAD4.4* | MZ747503 | FQGHH | HAWAH | HAAHH |
|  | *PfFAD4.5* | MZ747507 | FQGHH | HSWAH | HAAHH |
| Δ7 desaturase | *PfADS3.1* | MZ747466 | — | HRHHH | HNNHH |
|  | *PfADS3.2* | MZ747467 | — | HRHHH | HNNHH |
| Δ9 desaturase | *PfFAB2.1* | MZ747468 | EENRHG | DEKRHE | — |
|  | *PfFAB2.2* | MZ747470 | EENRHG | DEKRHE | — |
|  | *PfFAB2.3* | MZ747477 | EENRHG | DEKRHE | — |
|  | *PfFAB2.4* | MZ747479 | EENRHG | DEKRHE | — |
|  | *PfFAB2.5* | MZ747482 | EENRHG | DEKRHE | — |
|  | *PfFAB2.6* | MZ747484 | EENRHG | DEKRHE | — |
|  | *PfFAB2.7* | MZ747485 | EENRHG | DEKRHE | — |
|  | *PfFAB2.8* | MZ747486 | — | DEKRHE | — |
|  | *PfFAB2.9* | MZ747491 | EENRHG | DEKRHE | — |
|  | *PfFAB2.10* | MZ747493 | EENRHG | DEKRHE | — |
|  | *PfFAB2.11* | MZ747497 | EENRHG | DEKRHE | — |
|  | *PfFAB2.12* | MZ747505 | EENRHG | DEKRHE | — |
|  | *PfFAB2.13* | MZ747506 | EENRHG | DEKRHE | — |
| Δ12 desaturase | *PfFAD2.1* | MZ747489 | HECGHH | HRRHH | — |
|  | *PfFAD2.2* | MZ747499 | HECGHH | HRRHH | HVAHH |
|  | *PfFAD2.3* | MZ747500 | HDCGHH | HRRHH | HVVHH |
|  | *PfFAD6.1* | MZ747487 | HDCAHK | HDRHH | HIPHH |
|  | *PfFAD6.2* | MZ747504 | HDCAHK | HDRHH | HIPHH |
| ω3 desaturase | *PfFAD3.1* | MZ747492 | HDCGHG | HRTHH | HVAHH |
|  | *PfFAD3.2* | MZ747469 | HDCGHG | HRTHH | HVAHH |
|  | *PfFAD7/8.4* | MZ747495 | TAVGHG | HRTHH | — |
|  | *PfFAD7/8.5* | MZ747502 | IAVGHG | HRTHH | — |
|  | *PfFAD7/8.1* | MZ747471 | HDCGHG | HRTHH | HVAHH |
|  | *PfFAD7/8.2* | MZ747480 | HDCGHG | HRTHH | HVAHH |
|  | *PfFAD7/8.3* | MZ747483 | HDCGHG | HRTHH | HVAHH |
| Front-end desaturase | *PfSLD1.1* | MZ747475 | HDSGHY | HNAHH | QIEHH |
|  | *PfSLD1.2* | MZ747476 | HDSGHY | HNAHH | QLEHH |
|  | *PfSLD1.3* | MZ747478 | HDSGHY | HNAHH | QLEHH |
|  | *PfSLD1.4* | MZ747481 | HDSGHY | HNAHHIA | QIEHH |
|  | *PfSLD1.5* | MZ747498 | HDSGHY | HNAHH | — |
|  | *PfSLD1.6* | MZ747501 | HDSGHY | HNAHH | QIEHH |
|  | *PfDES1.1* | MZ747472 | HELSHN | HLEHH | HNEHH |
|  | *PfDES1.2* | MZ747473 | HELSHN | HLEHH | HNEHH |
|  | *PfDES1.3* | MZ747490 | HELSHN | HLEHH | HNEHH |
|  | *PfDES1.4* | MZ747494 | HELSHN | HLEHH | — |

**TABLE | S5. The FPKM of *PfFAD* family genes**

| **Gene** | **Ro** | **St** | **Le** | **Fl** | **2D** | **5D** | **7D** | **10D** | **12D** | **16D** | **19D** |
| --- | --- | --- | --- | --- | --- | --- | --- | --- | --- | --- | --- |
| *PfFAD4.1* | 0.09 | 0 | 0 | 0 | 0 | 0 | 0 | 0 | 0 | 0 | 0 |
| *PfFAD4.2* | 0 | 0 | 0 | 0 | 0 | 0 | 0 | 0 | 0 | 0 | 0 |
| *PfFAD4.3* | 0 | 0 | 0 | 0.72 | 0.34 | 0.46 | 2.73 | 7.84 | 0.7 | 0.21 | 0 |
| *PfFAD4.4* | 0.15 | 0 | 50.69 | 14.96 | 0.98 | 1.44 | 5.3 | 2.3 | 1.08 | 1.25 | 0.14 |
| *PfFAD4.5* | 0 | 0 | 0 | 0 | 0 | 0 | 0 | 0 | 0 | 0 | 0 |
| *PfADS3.1* | 4.74 | 14.99 | 49.61 | 39.78 | 19.28 | 19.76 | 18.06 | 21.34 | 31.88 | 37.4 | 21.48 |
| *PfADS3.2* | 1.24 | 10.8 | 88.04 | 21.44 | 9.36 | 8.42 | 8.52 | 8.3 | 13.01 | 10.6 | 9.84 |
| *PfFAB2.1* | 0 | 0.33 | 0 | 18.23 | 1.78 | 1.15 | 0.15 | 0 | 0 | 0 | 0 |
| *PfFAB2.2* | 0 | 9.39 | 0 | 0 | 0 | 0 | 0 | 0 | 0 | 0 | 0 |
| *PfFAB2.3* | 2.08 | 0.29 | 0 | 0.85 | 1.48 | 1.39 | 1.44 | 4.26 | 14.9 | 13.95 | 18.59 |
| *PfFAB2.4* | 5.96 | 13.54 | 18.52 | 7.56 | 21.55 | 9.51 | 9.04 | 21.7 | 20.15 | 8.85 | 9.2 |
| *PfFAB2.5* | 0.54 | 0.44 | 0.29 | 0.56 | 1.36 | 3.68 | 2.6 | 6.58 | 19.57 | 26.08 | 20.67 |
| *PfFAB2.6* | 0 | 0 | 0 | 35.61 | 0.36 | 0 | 0 | 0 | 0 | 0 | 0 |
| *PfFAB2.7* | 226.16 | 0.22 | 0 | 0.07 | 0.87 | 0.13 | 25.69 | 701.56 | 481.04 | 53.84 | 6.99 |
| *PfFAB2.8* | 0 | 0 | 0 | 0 | 0 | 0 | 0 | 0 | 0 | 0 | 0 |
| *PfFAB2.9* | 5.28 | 16.72 | 22.09 | 8.09 | 19.9 | 13.46 | 11.34 | 29.13 | 31.94 | 11.75 | 8.66 |
| *PfFAB2.10* | 14.99 | 19.75 | 25.55 | 50.99 | 36.32 | 24.22 | 65.95 | 224.89 | 227.3 | 216.25 | 141.07 |
| *PfFAB2.11* | 5.11 | 0.59 | 23.44 | 13.64 | 8.39 | 4.5 | 110.14 | 1343.75 | 1362.62 | 1198.33 | 834.09 |
| *PfFAB2.12* | 8.88 | 9.87 | 7.88 | 29.6 | 20.04 | 14.88 | 29.84 | 89.74 | 96.1 | 107.82 | 77.84 |
| *PfFAB2.13* | 3.32 | 0.1 | 5.3 | 8.16 | 7.71 | 2.66 | 97.57 | 1254.46 | 1164.44 | 956.27 | 518.79 |
| *PfFAD2.1* | 96.33 | 110.84 | 21.06 | 479.93 | 195.73 | 114.32 | 300.31 | 1221.82 | 1478.1 | 2207.83 | 3191.01 |
| *PfFAD2.2* | 77.54 | 179.1 | 27.17 | 522.37 | 160.97 | 68.41 | 169.45 | 1174.58 | 1466.69 | 2251.41 | 3093.63 |
| *PfFAD2.3* | 0 | 0 | 0 | 0 | 0 | 0 | 0 | 0 | 0 | 0 | 0 |
| *PfFAD6.1* | 19.87 | 42.2 | 60.15 | 36.54 | 20.04 | 21.35 | 14.55 | 12.37 | 8.19 | 4.65 | 1.4 |
| *PfFAD6.2* | 17.07 | 39.52 | 36.05 | 27.35 | 25.53 | 29.27 | 14.05 | 8.71 | 6.96 | 2.66 | 2 |
| *PfFAD3.1* | 10.4 | 10.15 | 7.53 | 44.27 | 45.14 | 37.57 | 70.03 | 1063.92 | 1635.18 | 2643.36 | 1632.98 |
| *PfFAD3.2* | 11.46 | 10.12 | 5.69 | 64.91 | 77.81 | 122.9 | 102.34 | 1260.04 | 1936.75 | 2464.93 | 794.66 |
| *PfFAD7/8.1* | 7.35 | 11.65 | 25.54 | 21 | 31.6 | 31.9 | 47.35 | 26.94 | 35.98 | 73.22 | 16.14 |
| *PfFAD7/8.2* | 11.1 | 14.78 | 20.64 | 23.14 | 40.5 | 34.11 | 61 | 35.83 | 34.99 | 59.39 | 15.36 |
| *PfFAD7/8.3* | 8.16 | 18.44 | 89.07 | 37.87 | 25.08 | 26.66 | 12.91 | 2.45 | 2.16 | 1.48 | 0.2 |
| *PfFAD7/8.4* | 9.53 | 5.08 | 11.11 | 11.85 | 15.8 | 11.71 | 13.98 | 19.99 | 13.2 | 15.81 | 21.37 |
| *PfFAD7/8.5* | 10.5 | 7.02 | 3.66 | 11.7 | 12.57 | 10.12 | 14.73 | 12.9 | 8.06 | 11.71 | 17.7 |
| *PfSLD1.1* | 6.92 | 2.13 | 10.5 | 47.75 | 36.2 | 53.15 | 50.66 | 18.81 | 12.02 | 25.08 | 4.55 |
| *PfSLD1.2* | 7.24 | 11.99 | 7.09 | 11.69 | 27.11 | 16.73 | 10 | 10.03 | 11.67 | 35.79 | 63.05 |
| *PfSLD1.3* | 6.02 | 20.88 | 0.09 | 2.48 | 35.36 | 25.79 | 10.35 | 15.86 | 5.89 | 0.72 | 0.28 |
| *PfSLD1.4* | 16.39 | 25.9 | 11.41 | 24.63 | 51.14 | 40.82 | 23.25 | 18.46 | 28.6 | 58.32 | 105.28 |
| *PfSLD1.5* | 5.47 | 2.05 | 5.38 | 15.73 | 19.12 | 32.64 | 45.28 | 20.15 | 11.53 | 15.36 | 1.79 |
| *PfSLD1.6* | 4.9 | 12.47 | 0.68 | 3.54 | 26.83 | 14.47 | 9.86 | 15.3 | 7.78 | 0 | 0.27 |
| *PfDES1.1* | 5.65 | 5.41 | 1.23 | 6.44 | 25.13 | 16.8 | 8.73 | 6.59 | 4.23 | 2.15 | 3.42 |
| *PfDES1.2* | 1.9 | 2.51 | 0.25 | 5.17 | 9.77 | 5.88 | 4.73 | 5.26 | 7.46 | 6.13 | 3.42 |
| *PfDES1.3* | 2.44 | 5.45 | 0 | 5.3 | 5.47 | 2.55 | 0.27 | 2.83 | 3.19 | 2.21 | 1.21 |
| *PfDES1.4* | 2.75 | 4.79 | 0.1 | 5.16 | 6.67 | 3.64 | 2.98 | 6.22 | 6.13 | 2.44 | 4.02 |

Note: Ro, roots; St, stems; Le, leaves; Fl, flowers; 2D–20D means seeds at 2 to 20 days after flowering


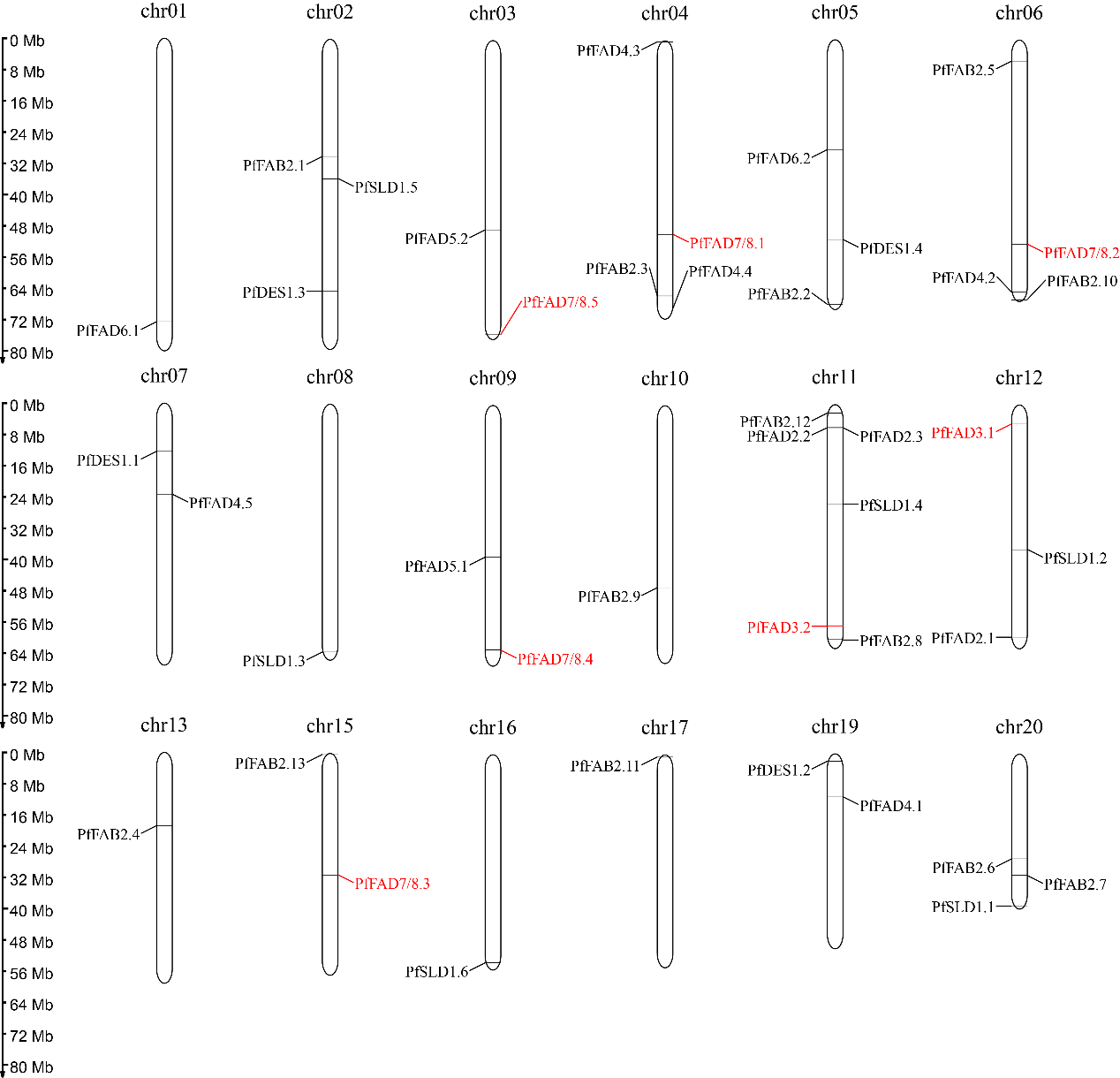


**FIGURE | S1 Chromosomal distribution of *PfFAD* genes was drawn with Map Chart v 2.2.**

**
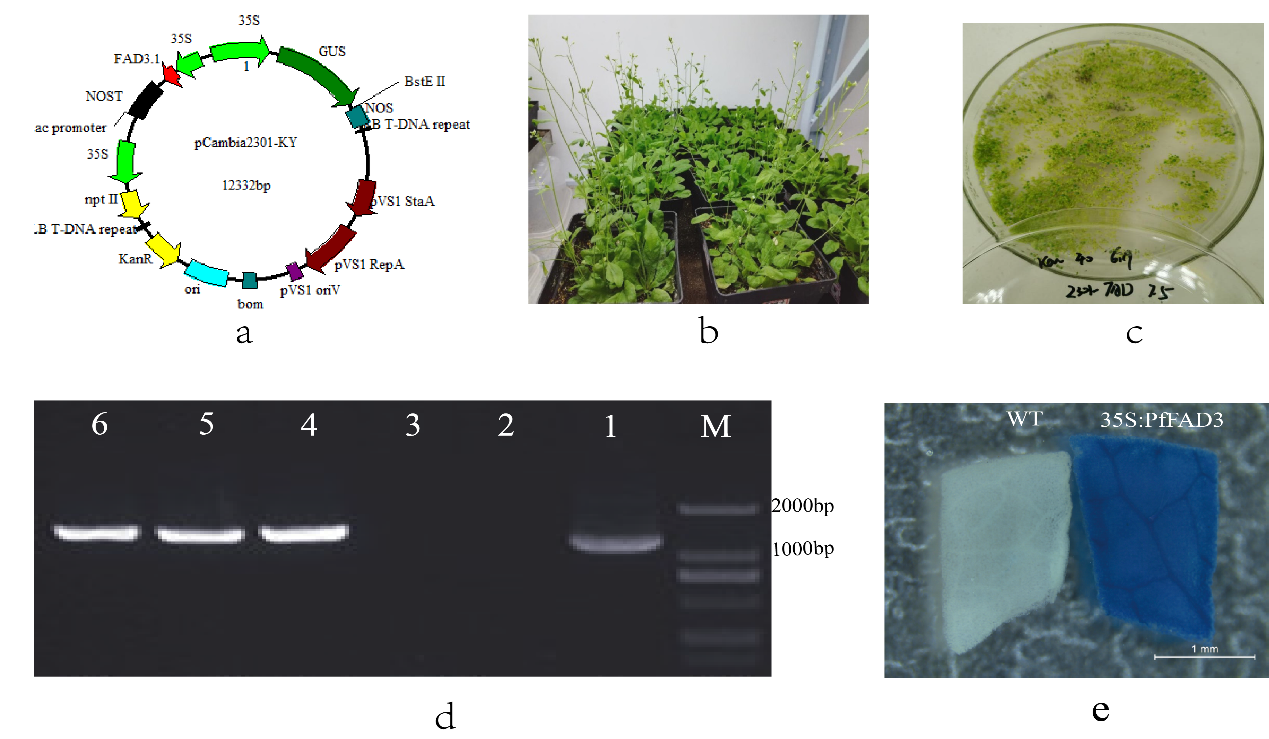
**

**FIGURE | S2 Transgenic Arabidopsis with *PfFAD3.1* gene and its identification.** (a) pCambia2301-FAD3.1 vector. (b) pCambia2301-FAD3.1 vector to introduced into *A. thaliana.* (c) Kanamycin Screening. (d) PCR identification. M: Mark D2000, 1-6 means samples pCAMBIA2301-PfFAD3.1vector, ddH_2_O, WT, 35S:PfFAD3.1-1, 35S:PfFAD3.1-5, 35S:PfFAD3.1-6 respectively. (e) GUS staining.


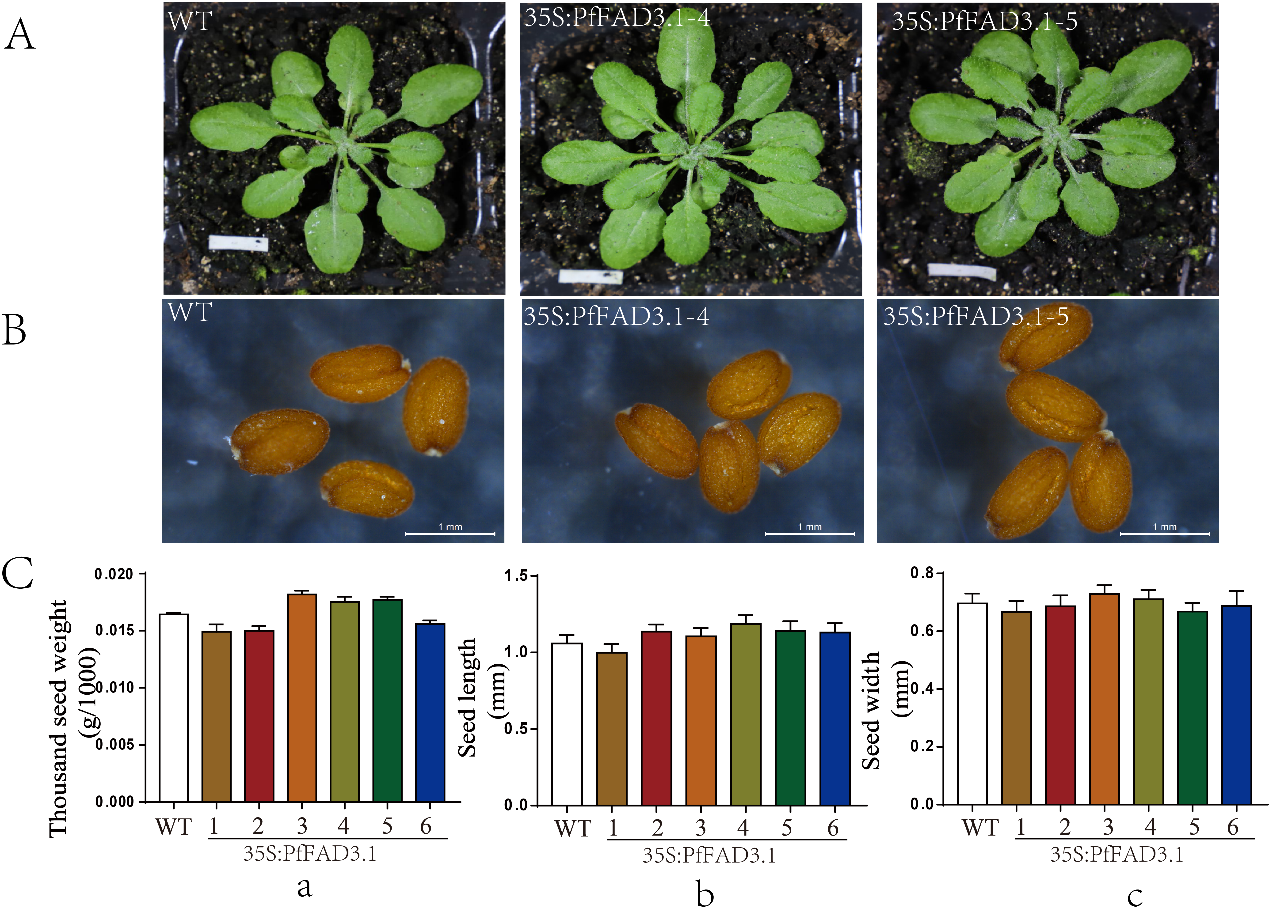


**FIGURE | S3 Plant development and seed characteristics of transgenic Arabidopsis with *PfFAD3.1* gene.** (A)Transgenic Arabidopsis line, (B) Seeds of transgenic Arabidopsis and (C)Thousand seed weight (a), seed length(b) and seed width (c)of transgenic Arabidopsis

**FIGURE | S4. Multiple sequence alignment of *FAD3* gene in *Perilla***
